# Log‐ratio analysis of microbiome data with many zeroes is library size dependent

**DOI:** 10.1111/1755-0998.13391

**Published:** 2021-05-03

**Authors:** Dennis E. te Beest, Els H. Nijhuis, Tim W. R. Möhlmann, Cajo J. F. ter Braak

**Affiliations:** ^1^ Biometris Wageningen University & Research Wageningen The Netherlands; ^2^ Biointeractions and Plant Health Wageningen University & Research Wageningen The Netherlands; ^3^ Laboratory of Entomology Wageningen University & Research Wageningen The Netherlands

**Keywords:** log‐ratio analysis, microbiome, multivariate statistics, zero inflation

## Abstract

Microbiome composition data collected through amplicon sequencing are count data on taxa in which the total count per sample (the library size) is an artefact of the sequencing platform, and as a result, such data are compositional. To avoid library size dependency, one common way of analysing multivariate compositional data is to perform a principal component analysis (PCA) on data transformed with the centred log‐ratio, hereafter called a log‐ratio PCA. Two aspects typical of amplicon sequencing data are the large differences in library size and the large number of zeroes. In this study, we show on real data and by simulation that, applied to data that combine these two aspects, log‐ratio PCA is nevertheless heavily dependent on the library size. This leads to a reduction in power when testing against any explanatory variable in log‐ratio redundancy analysis. If there is additionally a correlation between the library size and the explanatory variable, then the type 1 error becomes inflated. We explore putative solutions to this problem.

## 1 INTRODUCTION

Microbiome composition data collected through amplicon sequencing are count data on taxa in which the total count per sample (the library size) is a technical, ill‐understood artefact, which carries no biological information, and as a result, such data are compositional. Some people have advocated the use of compositional data analyses in analysing such data (Gloor et al., 2017; Tsilimigras & Fodor, 2016). For multivariate analysis, this implies transforming the data with the centred log‐ratio transformation (clr) followed by a standard least‐squares method such as principal component analysis (PCA). Equivalently, the data (counts or proportions) are logarithmically transformed and double‐centred, followed by a PCA. This is often called log‐ratio PCA or log‐ratio analysis (Aitchison, 1983; Greenacre, 2018). Mathematically, this is a solid approach when there are no zeroes, as it takes care of the arbitrary total per sample by only analysing log‐ratios (Greenacre, 2018). However, with zeroes, a pseudo‐count must be added before taking the log transformation.

Two aspects typical for amplicon sequencing data complicate the use of log‐ratio PCA: the high amount of zeroes combined with a large variability in the library size. In this study, we show that using log‐ratio PCA on such data has the unexpected and unwanted consequence that the library size again influences the analysis. In an unconstrained analysis (PCA), it is possible that the 1st or 2nd axes primarily display the library size. In a constrained analysis (e.g. log‐ratio redundancy analysis (RDA)) (ter Braak, 1994; van der Wollenberg, 1977), this effect complicates the assessment of significance of explanatory variables.

The primary aim of this study was to make people aware of this problem of using log‐ratio analysis and the clr transformation on amplicon sequencing data. We provide a mathematical explanation and illustrate the issue with simulated data and two amplicon sequencing data examples. We additionally explore some putative solutions.

## 2 MATERIALS AND METHODS

### 2.1 Log‐ratio PCA

With the aim to compare samples, log‐ratio PCA decomposes **Y**, a matrix that contains compositional data with I samples (rows) and J taxa (columns), to a set of principal axes (Aitchison, 1983; Greenacre, 2018). We define $L=\{l_{\mathrm{ij}}\}$ as the log of **Y**, **r** as the marginal mean of the rows of **L** and **c** as the marginal mean of the columns of **L**. The log‐ratio transformation (clr) is defined as $log(y_{i}/g\left(y_{i}\right))$ (where $y_{i}$ is the i‐th row of **Y** and g() is the geometric mean), which is equivalent to $l_{\mathrm{ij}}‐r_{i}$. Given that in a decomposition to principal axes we also need to centre by taxa (columns), a log‐ratio PCA involves double centring of **L**.

(1) $$s_{\mathrm{ij}}=l_{\mathrm{ij}}‐r_{i}‐c_{j}+l_{..}$$

where $l_{..}$ stands for the global mean of **L**. The centred matrix $S=\{s_{\mathrm{ij}}\}$ can be decomposed with a singular value decomposition (SVD):

(2) $$S=U\Sigma V^{T}$$

Matrix U, of size I×K, contains the ‘sample scores’, where K stands for the number of latent dimensions. Matrix V is of size J×K and contains the ‘taxon scores’. Matrix Σ is diagonal matrix with singular values (Greenacre, 2012, 2018). The main focus in our analyses is on comparing the sample scores.

### 2.2 Zeroes lead to library size dependence: Mathematical explanation

A large number of zeroes combined with a large variability in library size, and thus in r, create a problem for log‐ratio PCA. For count data, it is common to add a pseudo‐count of 1. This preserves the zeroes and the sparsity of the data, and avoids needing to take the log of zero, but it destroys the proportionality to the library size, which is key to log‐ratio analysis, particularly for low count values, including zeroes. After row centring (i.e. deducting r), taxa with many zeroes (and/or many ones and/or twos) will now primarily contain elements of r, in particular, for zero counts $s_{\mathrm{ij}}\approx ‐r_{i}$. All taxa with many zero values (taxa with a low prevalence, rare taxa, for short) or with very low counts are therefore positively correlated amongst one another and all are negatively correlated with r. If many such taxa exist, and there is a substantial variability in r, a considerable part of the variance of S is related to r. Both the variance in S that is explained by r and the correlation between r and S for rare taxa increase as both the variability in r and the number of zeroes increase.

Given enough variability in r and enough zeroes in the data, a log‐ratio PCA identifies this artefact as a prominent effect. In this situation, the effect of r is in competition with other effects and may either influence any of the principal axes or even completely dominate the first axis. In a constrained analysis, for example log‐ratio RDA, an explanatory variable that happens to be correlated with r is likely to be judged significant in permutation testing, even if it is unrelated to the taxa data (type 1 error inflation). By contrast, there will be little power to detect explanatory variables that are uncorrelated with r, but do influence the microbiome.

We call the problem informally ‘library size dependence’ and the cause ‘variability in library size’, although the formal cause is variability in **r**. It is important to note that data with an equal library size or equalized library size (rarefaction) may also show variability in **r** (Figure S1). In most cases, the library size and r are correlated, and if a correlation exists between **S** and the library size, there is likely also correlation between **S** and **r**. Note that the problem we describe is not purely related to the amount of zeroes; it can also be ascribed to a lack of variability in taxon abundance. If the variance of a particular taxon is low, then, after double centring, its variance is largely explained by **r**. In practice, a low variance is primarily observed for rare taxa.

### 2.3 Diagnostics

We propose two diagnostics to assess library size dependency in log‐ratio PCA of sparse data. We cannot exclude that other data characteristics can cause the patterns described below, but in the context of a log‐ratio PCA applied to amplicon sequencing data it is likely that a fit is influenced by the library size via row centring if these patterns arise.

The first diagnostic is to calculate the correlation between each column of S and r (hereafter the correlations are collectively denoted by $\rho_{\mathrm{Sr}}$) and to plot this correlation against the log of the mean abundance per taxon (i.e. the log of marginal column mean of Y). A negative value of $\rho_{\mathrm{Sr}}$ for a low abundance taxon suggests that this taxon primarily contains elements of ‐r. If S contains the effect of r, we expect that the low abundance taxa have a strong negative correlation with r. Library size dependence is diagnosed if the graph of $\rho_{\mathrm{Sr}}$ against the log taxon mean shows an increasing trend starting from a low y‐axis value (e.g. −0.5; see examples). This does not necessarily mean the 1st or 2nd PCA axis is influenced by r, and its effect may also be expressed on a subsequent axis. If this trend is absent, then there is no dependence on r or the library size. Note that the correlation diagnostics can be used on any clr‐transformed matrix and is not specific for log‐ratio PCA.

The second diagnostic we suggest is specific for log‐ratio PCA; it is a plot of the (log) contribution of each taxon to a particular principal axis against the log of the mean abundance per taxon (i.e. the log of marginal column mean of Y). The contribution of a taxon to an axis can be quantified with the square of its value in V (Greenacre, 2013a, 2013b), which is output of the earlier described SVD (equation 2). A PCA axis is suspicious if all low abundance taxa have a relatively high and about equal contribution. In such a case, these low abundance taxa are likely contributing due to their negative correlation with r and they are contributing to an axis that primarily contains the effect of r. As taxon abundance and variance increase, the correlation with r reduces and the contribution drops. The most abundant taxa tend to have few zero values and are thus unaffected by r. In extreme cases, the resulting pattern is V‐shaped. By contrast, if the mean contribution is either a gradually increasing (on the log scale) with taxon abundance or highly variable around a constant, the PCA axis is unsuspected.

Another possible diagnostic is to fit a log‐ratio RDA with r as the constraining variable and estimate how much variance in S is related to r. The problem with this diagnostic is that it is unclear what percentage of r related variance is low or high, that is we have nothing to compare with. It also possible to quantify the amount of variance in S per taxon that can be explained by r (with $\rho_{\mathrm{Sr}}^{2}$); this is addressed with the first diagnostic.

### 2.4 Examples

One example in this study is based on simulation, and two examples are based on amplicon sequencing data. The aim of the simulation is to illustrate what may go wrong with log‐ratio PCA. To make transparent how the row centring problem arises, we opt for a relatively simple simulation setting that allows us to assess the effect of a large number of zeroes with a large variation in the library size and, optionally, a correlation between x and r. The two data examples demonstrate how the row centring problem manifests itself in amplicon sequencing data.

#### 2.4.1 Simulation

In the simulation, we draw a matrix of counts, **Y**, with I samples and J taxa. By default, we set I=50 and J=500. As microbiome data commonly show overdispersion compared with the Poisson distribution (McMurdie & Holmes, 2014), Matrix **Y** is sampled from a negative binomial distribution with mean $\mu_{\mathrm{ij}}$ and variance $\mu_{\mathrm{ij}}+\mu_{\mathrm{ij}}^{2}$. We set the expectation $\mu_{\mathrm{ij}}$ with a log‐linear model: $log\left(\mu_{\mathrm{ij}}\right)=a_{i}+t_{j}+b_{j}x_{i}$, where $a_{i}$ reflects the library size and is drawn according to $a_{i}:N\left(0,\sigma_{a}\right)$, $t_{j}$ reflects the overall abundance of taxon j and is drawn according to $t_{j}:N\left(0,\sigma_{t}\right)$, and $x_{i}$ represents a binary (0/1) variable representing two treatment groups of equal size with $b_{j}$ the treatment effect on taxon j. By default, we set $\sigma_{t}=2$, and we set $\sigma_{a}$ to either 0, 0.5 or 1 so as to study the effects of library size. At random, 100 out of 500 taxa are made differentially abundant, which are at random with equal probability either up‐ or downregulated by setting $b_{j}$ equal to b and ‐b, respectively; for the remaining taxa, $b_{j}=0$. Unless stated otherwise, taxa present in less than 5 samples are removed.

It is of interest to see how log‐ratio PCA performs if the library size is correlated with the treatment, for example if the samples from one treatment group tend to have a higher library size than the samples from the other treatment group. We simulate such scenario by incorporating a correlation between x and r. This is achieved by modelling x with a logistic function, according to $x_{i}:Bernoulli\left(g\frac{e^{\gamma a_{i}}}{1+e^{\gamma a_{i}}}\right)$. With parameter γ, we can set strength of the correlation ($\rho_{\mathrm{xr}}$). Parameter g is set for each simulated draw to ensure that the treatment groups are equal in size.

We first use the simulation model to demonstrate the diagnostics by simulating one data set per level of library size variability, that is $\sigma_{a}$ = 0, 0.5 and 1, in the situation without correlation between x and r, that is. γ=0. This results in example data sets with library sizes of, respectively, 2731 to 5842, 1215 to 13256, and 349 to 34907. After removing taxa with <5 occurrences, these examples contain 445, 441 and 458 taxa and 42%, 42% and 44% zeroes, respectively. The fold change for the differentially abundant taxa in these simulations is set to 3 (b = log(3)).

Next, we repeatedly simulate new data to estimate the type 1 error and power of a log‐ratio RDA to detect the effect of the treatment x at the nominal significance level of 0.05. Here, we explore two scenarios. First, we assess how variability in r affects the type 1 error and power by varying the fold change (in four steps from 1 to 2) for three levels of $\sigma_{a}$. In this scenario, there is no correlation between x and r (γ = 0). In a second scenario, we explore what effect the correlation between x and r has on the type 1 error by varying γ between 0 and 3. As this scenario concerns type 1 error, there is no treatment effect (fold change = 1, b = log(1)). With γ=2, the average (Pearson's) correlation across 2000 simulations between x and r is 0.23, 0.41 and 0.58 for, respectively, $\sigma_{a}$ of 0.25, 0.5 and 1. For a visualization of $\rho_{\mathrm{ax}}$ and $\rho_{\mathrm{rx}}$ for various values of γ, we refer to supplemental Figure S18. In the power and type 1 error simulations, we also explore some putative solutions and assess how robust these are to the studied data characteristics. These solutions consist of alternative versions of log‐ratio PCA and closely related methods. Details on these methods are available in the supplementary information.

#### 2.4.2 Biting midges data

In the first real data example, we examine a data set of 191 observations on laboratory‐reared biting midges. Each observation contains the pooled abdomens of 5 adult female biting midges that were fed for a period of time after hatching on sugar water supplemented with or without antibiotics to affect the gut microbiome. In total, 86 pools contained biting midges that received antibiotics and 105 pools received no antibiotics. Per pool fragment DNA was isolated, fragments of 16S were (amplified and) sequenced (Illumina MiSeq) and grouped into amplicon sequence variants (ASVs). For more information, we refer to the original publication (Möhlmann et al., 2020).

The original publication analysed multiple biting midge species; here, we only use the *Culicoides nubeculosus* samples. In the original study, only the samples were used with biting midges fed on sugar water for a period of 6 days, as this gave the best indication of the effect of antibiotics. For illustration purpose, we use all sequenced samples for this species that were collected during the course of the experiment (data from 2nd to 11th day feeding on sugar water with and without antibiotics). Analogous to the simulated example, we call the treatment variable x.

**FIGURE 1:**
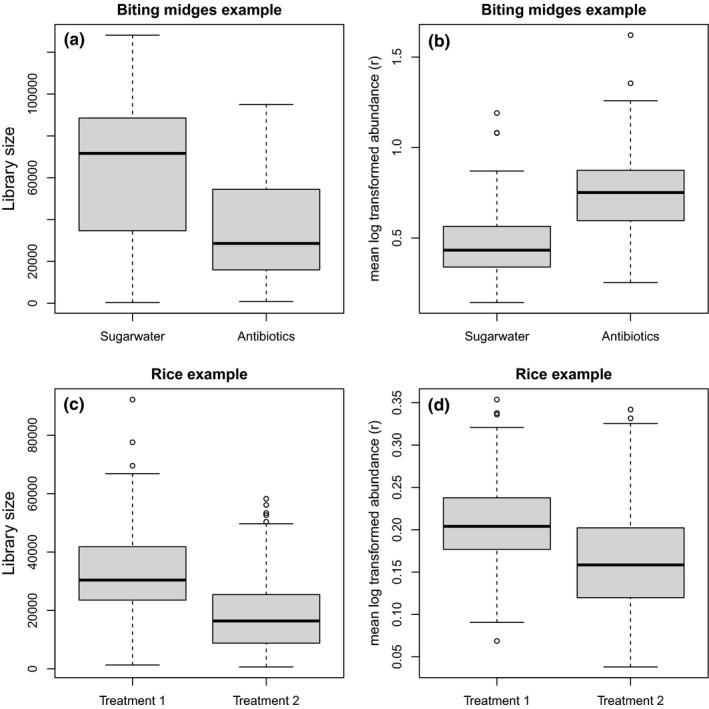
The library size (a and c) and the mean r (b and d) per treatment for both example data sets. In both examples, the library size and r are correlated with the treatment (x)

We removed ASVs that were absent in 10 or more samples, leaving 155 taxa, containing 85% zeroes. The library size varies from 335 to 128.175 reads. Both the library size and r are correlated with the treatment variable (Figure 1), but with opposite signs. The (Pearson) correlation between x and r is 0.54 in absolute value. See supplemental Figure S1 for the correlation between r and the treatment variable after rarefaction.

#### 2.4.3 Rice data

In the second real data example, we examine a data set about the root‐associated microbiome of 296 rice cultivars cultivated under field conditions. Each cultivar was grown with sufficient (control) and insufficient water (drought), giving 592 observations. Each observation contains the material of three pooled replicates. Per observation, DNA was isolated, and fragments of 18S were (amplified and) sequenced (Illumina MiSeq) and clustered into operational taxonomic unit (OTUs). Analogous to the simulated example, we call the treatment variable x. For further details, we refer to Andreo‐Jiménez; Andreo‐Jiménez et al., (2019).

Taxa that were absent in 10 or more samples were removed, leaving 650 taxa, which together contained 92% zeroes. The library size varies from 651 to 92.224 reads. Both the library size and r are correlated with the treatment variable (Figure 1). The (Pearson) correlation between x and r is 0.40 in absolute value. See supplemental Figure 1 for the correlation between r and the treatment variable after rarefaction.

### 2.5 Software

Log‐ratio PCA was carried out using the function dudi.pca from R package ade4 Dray and Dufour (2007) using a double‐centred log‐transformed count matrix as input. For the log‐ratio RDA (constrained analysis), we subsequently used the function pcaiv, and testing was done with randtest (both ade4), which performs a Monte Carlo permutation test (999 permutations). The testing was done on the percentage of explained variance, that is constrained inertia in ade4.

## 3 RESULTS

### 3.1 Diagnostics

#### 3.1.1 Simulated examples

**FIGURE 2:**
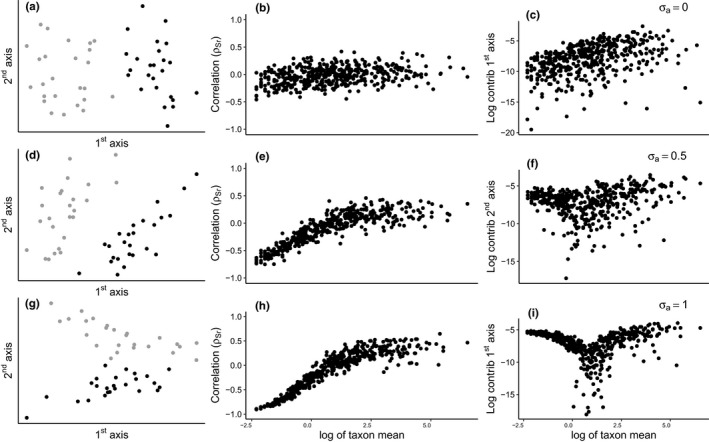
Simulated data. Log‐ratio PCA and diagnostics (columns) for three levels of library size variability (rows: $\sigma_{a}$=0, $\sigma_{a}$=0.5, $\sigma_{a}$=1). The first column (a, d, g) displays the simulated observations on the 1st and 2nd principal axes, and colours indicate treatment groups. The second column (b, e, h) displays the correlation between **S** (clr‐transformed abundances) and r, and the third column (c, f, i) displays the contribution of a taxon versus its log mean abundance. For $\sigma_{a}=1$, the 1st axis contains the effect of r and the effect of x is pushed to the 2nd axis

The simulated data examples illustrate how log‐ratio PCA is influenced by variability in library size in the presence of zeroes (Figure 2). If the variation is low ($\sigma_{a}=0$), the samples of the two treatment groups are clearly separated along the 1st PCA axis. There is no strong trend in $\rho_{\mathrm{Sr}}$ against the log taxon mean (Figure 2a–c), and the taxon contribution increases on average with taxon abundance. This demonstrates that for this scenario log‐ratio PCA performs well, despite the presence of a large number of zeroes (42%).

If the variation in library size is increased ($\sigma_{a}=0.5$, Figure 2d–f), the effect of r starts to compete with x. The first axis still largely contains the effect of x, but r is affecting the 2D sample configuration. We see a clear trend in $\rho_{\mathrm{Sr}}$ with taxon abundance, and the contributions to the 2nd axis display relatively high contributions for the low abundance taxa. If we further increase the variation in library size ($\sigma_{a}=1$, Figure 2g–i), the effect of x is pushed to the 2nd axis. The 1st axis now reflects r and thus the library size. The trend in correlations is more pronounced, with many abundant taxa having positive correlation (up to 0.5), so that the contribution plot shows a V‐shaped pattern. See supplemental Figures S2–S14 for the performance of the putative solutions on the simulated example.

#### 3.1.2 Data examples

**FIGURE 3:**
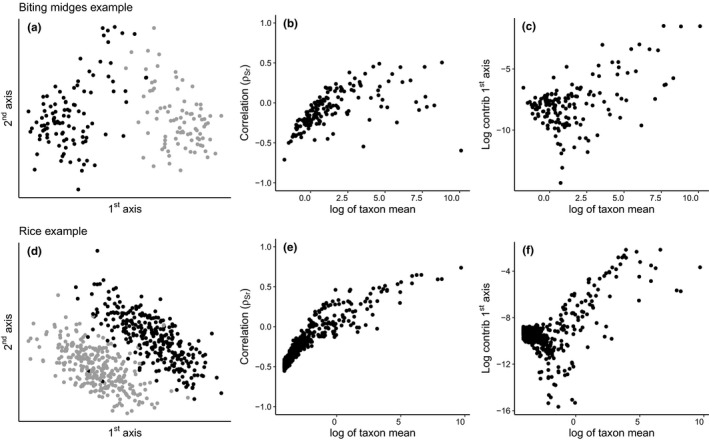
Log‐ratio PCA and diagnostics (columns) for the real data examples. The first column (a and d) displays the observations on the 1st and 2nd principal axes, and colours indicate treatment groups. The second column (b and e) displays the correlation between **S** (transformed abundances) and r. The third column (c and f) displays the log contribution to the 1st axis per taxon versus its log mean abundance. The negative correlations and the relatively high and similar contributions amongst the low abundance taxa suggest there is an issue with row centring (and thus with log‐ratio PCA) for both data examples

In both real data examples, we see a good separation of the treatment groups in a two‐dimensional log‐ratio PCA, suggesting the treatment has a strong effect (Figure 3a and d). For the biting midges example, this effect is on the 1st axis. For the rice example, this effect seems to be tilted. For both data examples, we see a clear trend in $\rho_{\mathrm{Sr}}$ against taxon abundance (Figure 3b and e) and a relatively high and about equal contribution amongst the low abundance taxa (Figure 3c and f). These patterns are similar to what we observed in the simulated example. These results suggest that the 1st axis, at least partly, contains the effect of r.

Given the correlation between x and r in these data sets (Figure 1), it is likely that in both data examples the 1st axis contains both the effect of x and r. In the rice example, it is possible that the tilting of the effect is caused by the effect of r (similar to the simulated example with $\sigma_{a}=0.5$, Figure 2D). For the diagnostics, it is clear that the log‐ratio PCA results are, at least partly, influenced by the library size. See supplemental Figures 2, 3, 4, 5, 6 for the performance of the putative solutions on the data examples and supplemental Figures S15–S17 for additional data examples.

**FIGURE 4:**
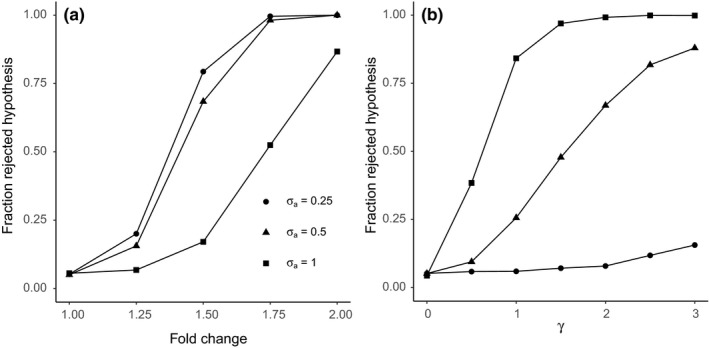
Rejection rate (number of *p*‐values <.05 across 2000 simulations) in testing the treatment effect using log‐ratio RDA. In (A), the fold change is increased for several levels of $\sigma_{a}$ under independence of the treatment with the library size (γ=0). The type 1 error is controlled, but the power is reduced as $\sigma_{a}$ increases. In (B), there is no treatment effect (fold change is 1, b=0), but there is an increasing correlation between treatment and library size (set with γ≥0) for three levels of $\sigma_{a}$. The type 1 error is controlled for γ=0, but increases for higher values of γ and $\sigma_{a}$

**FIGURE 5:**
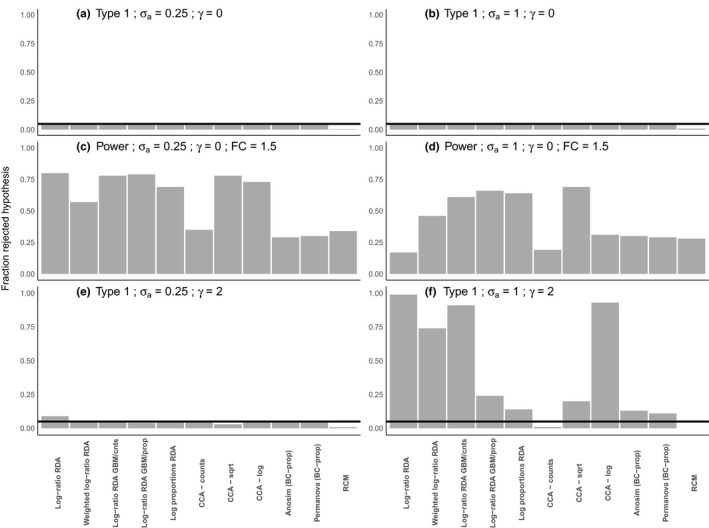
Type 1 and power for a set of methods closely related to log‐ratio RDA for two levels of $\sigma_{a}$. (a) and (b) display the type 1 error without correlation between x and r (γ=0). (c) and (d) display the power (fold change = 1.5) without correlation between x and r (γ=0). (E) and (F) display the type 1 error when there is a correlation between x and r (γ=2). For all methods (except RCM), the type 1 error and power were determined by counting the number of p‐values below 0.05 across 2000 simulations. For RCM, we did between 200 and 250 simulations, except for (F) where most estimations failed

**FIGURE 6:**
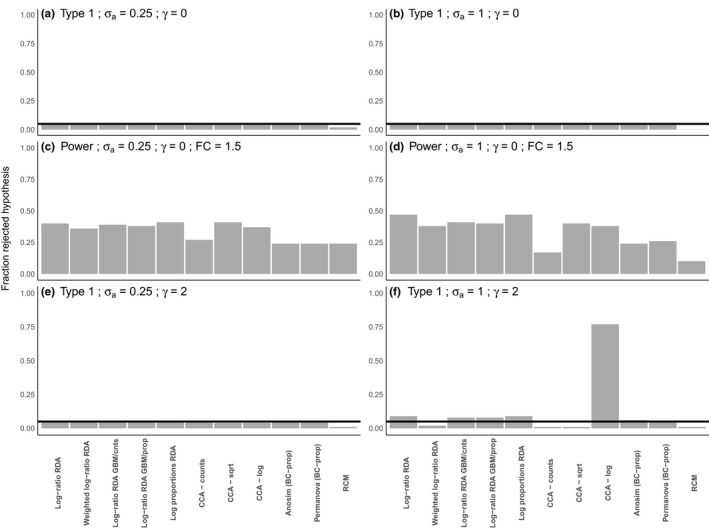
Type 1 and power for a set of methods closely related to log‐ratio RDA for two levels of $\sigma_{a}$. Compared to Figure 5, the simulated data were subject to an additional filtering step (see supplement for more information). (a) and (b) display the type 1 error without correlation between x and r (γ=0). (c) and (d) display the power (fold change =1.5) without correlation between x and r (γ=0). (e) and (f) display the type 1 error when there is a correlation between x and r (γ=2). For all methods (except RCM), the type 1 error and power were determined by counting the number of *p*‐values below .05 across 2000 simulations. For RCM, we did between 500 simulations

### 3.2 Power and Type 1 error

Without treatment effect (fold change =1, b=0) and with a treatment that is independent of the library size, log‐ratio RDA yields the correct type 1 error rate (0.05), irrespective of library size variability ($\sigma_{a}$; Figure 4a). With low‐to‐moderate library size variability ($\sigma_{a}\leq 0.5$), log‐ratio RDA has good power to detect the treatment effect. With a larger library size variability ($\sigma_{a}$ = 1), the power strongly decreases, for example with a fold change of 1.5 it decreases from about 0.75 at $\sigma_{a}=0.5$ to about 0.25 at $\sigma_{a}=1$. If the treatment is correlated with the library size (γ>0), log‐ratio RDA reasonably controls the type 1 error rate if the library size variability is low ($\sigma_{a}=0.25$). If the library size variability is moderate to large ($\sigma_{a}=0.5$ or 1), log‐ratio RDA shows strong type 1 error rate inflation, with error rates running close to 1, whereas the nominal level is 0.05 (Figure 4b).

Figure 5 compares type 1 error and power of some putative solutions (see supplementary information for details) with those of log‐ratio RDA. In the absence of correlation between treatment and library size ($\rho_{\mathrm{xr}}=0$, γ=0), all methods (including log‐ratio RDA) have a good control of the type 1 error, irrespectively of the amount of variation in library size ($\sigma_{a}$; Figure 5a and b, Figure 6a and b, Supplemental Table S1 and S2). However, the power of most putative solutions does not decrease as much with increasing library size variation ($\sigma_{a}$) as log‐ratio RDA does (Figure 5c and d). The methods log‐ratio RDA with geometric Bayesian multiplicative zero imputation (GMB, see supplement), log proportions RDA and canonical correspondence analysis (CCA, see supplement) on square‐rooted data are high‐ranked in terms of power with both low library size variation and high library size variation. After an additional filtering step, the drop in power for an increased $\sigma_{a}$ is minor or absent for all methods (Figure 6c and d, Supplemental Table S2). The improvement here is most notable for log‐ratio RDA.

With a correlation between treatment and library size (γ=2), the putative solutions control the type 1 error for moderate library size variation (Figure 5e), but show moderate‐to‐large type 1 error inflation (>0.10) for large library size variation ($\sigma_{a}=1$ (Figure 5f), with the exceptions of CCA on counts and RCM (row–column model; see supplement) that both perform badly in having a type 1 error rate that is too low (Figure 5f). Notably, log‐ratio RDA with GBM imputation on proportions shows less type 1 error inflation than log‐ratio RDA with GBM imputation on counts (Figure 5f). Part of the type 1 error inflation for all methods is caused by a difference in the number of zeroes between treatment groups of x that can occur as a result of $\rho_{\mathrm{xr}}$. In this scenario, the performance of all methods, but in particular of log‐ratio RDA, can be improved by filtering out low abundance taxa (Figure 6e and f, Supplemental Table S2).

## 4 DISCUSSION

Log‐ratio PCA is designed to give results that are library size‐independent. However, as we demonstrated mathematically and with examples based on simulated and real data, log‐ratio PCA becomes library size‐dependent, if there are many infrequent taxa (many zeroes) and library sizes differ largely. In this situation, the row centring used in log‐ratio PCA brings an effect of r (the row mean of the log‐transformed counts) in the clr‐transformed matrix. Note that this effect is irrespective of whether or not these infrequent taxa are genuine or due to sequencing noise or allocation error. This library size dependence is unexpected in the sense that, after applying the clr, the transformed matrix is free of the effect of the row totals for strictly positive data ($y_{\mathrm{ij}}>0$ for all i and j). We additionally demonstrate that library size variability causes a loss in power in detecting an effect of x with log‐ratio RDA. If there is additionally a correlation between treatment and the library size, the type 1 error for detecting the effect of x can be seriously inflated.

How serious is the issue in practice? It is important to note that we focus on fairly extreme scenarios in this study. Both example data sets have a high proportion of zeroes, large variation in library size and a correlation between treatment and library size. To some extent, this can be seen as a worst case scenario, but at the same time this is a realistic situation that may occur frequently with amplicon sequencing data. These data characteristics may also occur outside the field of amplicon sequencing, although we are unaware of such data. Note that RNASeq data are closely related, but have less zeroes and less variability in the library size. Our simulated data are also extreme, aimed at describing the issues that may arise.

Our main message is that one has to be careful when analysing data with the described characteristics with log‐ratio PCA. We provide two diagnostics. If these diagnostics display the patterns described in this study, additional actions are required. The most straightforward solution is stringent filtering out of low abundance or infrequent taxa. Note that, if a particular data set is less extreme in the described data characteristics than the data in this study, log‐ratio PCA will likely work and, in these cases, it is a powerful tool in analysing compositional data. We additionally explored various putative solutions (see also the supplementary information), some of which can also increase performance under the described circumstances.

There is a feature in the diagnostics that we do not fully understand mathematically, namely that many abundant taxa in situations with extreme library size variability show positive correlation ($\rho_{\mathrm{Sr}}$ up to 0.5) in the correlation diagnostic, resulting in extreme cases in a V‐shaped pattern in the contribution plot. These positive correlations occur in both the simulation and data examples (Figures 2 and 3) showing that the feature is real and not an artefact of our simulation. One possible explanation is that an effect of ‐r in low abundance taxa has to be compensated elsewhere, due to the zero‐sum constraint of the centred log‐ratio, resulting in positive correlations amongst high abundance taxa.

Although the focus of this study is on multivariate methods, there also consequences for other methods based on the clr. With high variation in library size and correlation between treatment and library size, low abundance clr‐transformed taxa will likely test significant in univariate analysis, even if there is no treatment effect, leading to type 1 error inflation. In case of graphical modelling with clr‐transformed taxa, we may detect spurious edges between low abundance taxa. The correlation diagnostic described in this study can also be used prior to such analyses.

To some extent, the large variability in library size and/or r and the large amount of zeroes are related to data quality. Currently, the variation in library size is ill‐understood, often not random, and it may even be correlated with a treatment variable, as in our examples. Future developments may lead to a better understanding of this variation and possibly, to more equal library sizes, which will reduce the problems we described.

## AUTHOR CONTRIBUTIONS

DETB, CJFTB and TWRM initialized the research. DETB performed analyses. DETB, CJFTB and EHN wrote the manuscript. All authors reviewed the manuscript.

## Supporting information

Supplementary Material

## Data Availability

Code and data are available on github.com/DennisBeest and in the supplement. The data of the midges example are also available from Mohlmann et al. (2020).
