# Supplementary material for: Log‐ratio analysis of microbiome data with many zeroes is library size dependent
Source: Mol Ecol Resour. 2021 May 3;21(6):1866–74. doi: 10.1111/1755-0998.13391 (PMC8360050; doi:10.1111/1755-0998.13391)
Supplement: Supplementary file 1 — Supplementary Material [file MEN-21-1866-s001.pdf]

Supplementary Information: Log-ratio analysis of microbiome data with many zeroes is library size dependent

Dennis E. te Beest[1a], Els H. Nijhuis[2], T.W.R. Möhlmann[3], Cajo J.F. ter Braak[1]

[1] Biometris, Wageningen University & Research, P.O. Box 16, 6700 AA, Wageningen, The Netherlands
[2] Biointeractions and Plant Health, Wageningen University & Research, P.O. Box 16, 6700 AA, Wageningen, The Netherlands
[3] Laboratory of Entomology, Wageningen University & Research, P.O. box 16, 6700 AA, Wageningen, The Netherlands
[a] Corresponding author

1 Aim of the supplementary material

In the supplementary material we explore a number of possible ways to analyze compositional data that have both a large number of zeroes and a large variability in library size and that may have a correlation between treatment and library size. We focus on log-ratio PCA/RDA and on some closely related alternatives, which are further described below. It is also possible to deal with such a data by conducting an additional preprocessing step, three of which we also describe below.

We explore the performance of these putative solutions on three examples described in the main text: one of the simulated data examples ($\sigma_a = 1$) and the two data examples. We then explore their power and type 1 error with further simulations. In the last section of the supplementary information we demonstrate the row centering problem of log-ratio PCA with three more examples based on publicly available data. For all methods, the type 1 error and power were determined by counting the number of p-values below 0.05 across 2000 simulations. Due to the computational burden of the row-column model (RCM) (Hawinkel et al., 2019), we had to reduce its number of simulations.

2 Preprocessing

2.1 Filtering

As described in the main text, log-ratio PCA has a problem with a large number of zeroes combined with a variability in row means of the log transformed

counts ($\mathbf{r}$). If we either reduce the number of zeroes or the variability in $\mathbf{r}$, we can expect a gain in performance. One way of reducing the number of zeroes in the data is by filtering out the low abundance taxa. Generally some amount of filtering is done anyway when preprocessing amplicon sequencing data to remove noise. At the same time, biologists typically are also interested in the low abundance taxa and prefer not to filter too much. The amount of filtering typically depends on the data at hand and how the reads are distributed across the taxa.

We explore the effect of reducing the number of zeroes with an additional filtering step for the data examples. For the rice data we create a filtered version by removing taxa that are absent in 150 or more samples (i.e. with ≥ 150 zeroes) and with a total count of at least 2000. This leaves 28 taxa that contain 32% zeroes. For the biting midges data we remove taxa that are absent in 75 or more samples and with total count of at least 2000. This leaves 17 taxa that contain 38% zeroes. For the simulated example with $\sigma_a = 1$ we remove taxa that are absent in 30 or more samples and with total count of at least 500. This leaves 63 taxa that contain 8% zeroes. We will refer to these data sets as the additionally filtered data, as opposed to the default data.

Another way of filtering is to remove samples with a low total library size. The aim of this step is to both remove zeroes and to equalize $\mathbf{r}$. If $\mathbf{r}$ is less variable, the amount of variance explained by $\mathbf{r}$ in $\mathbf{S}$ is reduced, which should have a positive effect on the performance. However, removing samples is typically quite wasteful. One problem with the approach is that we may not be removing samples at random, potentially causing a bias in the data. This is particularly evident when there is a correlation between some exploratory variable and the library size. For these reasons we do not explore the removal of samples further.

2.2 Rarefaction

Rarefaction equalizes the total number of counts (reads) per sample by randomly sampling the individual reads without replacement and is a popular preprocessing step in the field of amplicon sequencing. Rarefaction before applying log-ratio PCA could be part of a solution to the row centering problem addressed in this paper. Equalizing the library size is likely to make $\mathbf{r}$ less variable and with less variability in $\mathbf{r}$, log-ratio PCA should work better. It is important to note that equalizing the library size is not a guarantee to equalize $\mathbf{r}$. The top right of Figure 1 provides an example of this. In this particular example there is a shift in one of the dominant taxa and as a result the log transformation has a different effect on the two treatment.

Another issue with rarefaction is that we have to choose the lowest library size in the data as the rarefaction depth. This may involve throwing away a large part of the data (McMurdie & Holmes, 2014). If the lowest library size is high, the variability in $\mathbf{r}$ is likely low and problems with row centering are less pronounced. Alternatively, if the variability in $\mathbf{r}$ is high, the lowest library size is likely low. In this case rarefying the data to the lowest library size is quite wasteful and is generally not recommended. For these reasons we do not explore rarefaction further.

2.3 Zero imputation

Instead of adding a pseudo count to avoid the zeroes, some form of zero imputation can be used. We evaluate geometric Bayesian multiplicative imputation (GBM) (Martín-Fernández et al., 2015) as implemented in R package `zCompositions` (Palarea-Albaladejo & Martín-Fernández, 2015) with the function `cmultRepl` in combination with log-ratio PCA. This method is a common choice for zero imputation, also for microbiome data, see for example (Rivera-Pinto et al., 2018). We can either impute at the level of the counts, or first transform the data to proportions and then impute. We evaluate both these alternatives.

3 Alternative methods

3.1 Weighted log-ratio analysis

In weighted log-ratio PCA we give weights to the samples and taxa. There are various ways of setting these weights. In this supplement the row and column weights are set equal to the row and column means of $\mathbf{L}$ (i.e. the log-transformed counts), respectively. More details on weighted log-ratio PCA can be found in (Greenacre, 2018; Greenacre & Lewi, 2009).

The issue with row-centering in (unweighted) log-ratio PCA is particular present amongst the low abundance taxa. In weighted log-ratio PCA these low abundance taxa receive less weight, which means the effect of $\mathbf{r}$ is pushed to the background. Note however that the effect of $\mathbf{r}$ does not disappear from the weighted analysis; it may just be pushed away from the first axis to a later axis. One disadvantage of weighted log-ratio PCA is that high abundant taxa may receive a disproportionate amount of weight. As a result, a small number of taxa may dominate the fit. That is why we do not choose to row and column means of Y as weights.

3.2 Log proportions PCA

One possible alternative to log-ratio PCA is to analyze the data as log-proportions. Given the arbitrary library sizes, amplicon sequencing are often interpreted as proportions (i.e. total sum scaling). To reduce the influence of dominant taxa, it is common to take the log, resulting in: $s_{ij} = log(\frac{y_{ij}}{\sum_i y_{ij}} + c)$. Here c denotes a pseudo fraction that is needed to avoid zero. The resulting transformed matrix $\mathbf{S}$ can be decomposed with a `svd` (main text, equation (2)). Note that the pseudo fraction offers some control over the amount of shrinkage caused by the log. For a relatively high c, high proportions are pushed less to zero, and vice versa if c is low. In the examples and simulations we set c to 0.001. Note that c should not be chosen too low, as this increases the distances between the zeroes and the remaining data, essentially making the zeroes artificial outliers. If c is chosen too high, e.g. $c = 1$, we get close to the identity transformation (i.e. no transformation) because $log(x + 1) \approx x$ for small x.

One disadvantage of (log)-proportions is that we do not address the compositional nature of the data, i.e. we are analyzing relative data as if they are absolute. Whenever the absolute abundance of a particular taxon increases, the relative abundance of the other taxa has to decrease due to the constrained total sum, even when its absolute abundance did not change (Gloor et al., 2017). In a multivariate setting we are not looking at individual taxon; we are testing for a change in relative abundance across set of taxa that sum to 1. If there are no changes in absolute abundance in any of these taxa, there can also not be any spurious changes in relative abundance in any of the other taxa. This suggest that with (log)-proportions in a multivariate setting we have a correct null hypothesis and we can test for a change in composition as a result of some explanatory variable. A similar transformation that involves taking the square-root of the proportions (Hellinger) is not explored. Compared to the log, the square root shrinks high proportions less, and as a result we believe it is less suitable for examples analyzed in this paper.

3.3 Correspondence analysis and canonical correspondence analysis

Another alternative is correspondence analysis (CA) and its constrained form canonical correspondence analysis (CCA) (Greenacre, 2007; ter Braak & Verdonschot, 1995). CCA relates to CA as RDA relates to PCA. With CA (and CCA) we transform the data to deviations from expected values under row-column independence in a contingency table; these expected values are the product of the row and column sums of $\mathbf{Y}$ divided by the grand total of $\mathbf{Y}$. By this transformation, CA is expected to be insensitive to the library size (the row sum). However, perhaps some dependence may creep in again because CA also weighs these deviations based on the row and column sums. Specifically, we first divide the matrix of counts ($\mathbf{Y}$) by its grand total to obtain $\mathbf{P}$. Next we define $\mathbf{r}$ and $\mathbf{c}$ as the marginal sums of the rows and columns of $\mathbf{P}$, respectively. (Note that these $\mathbf{r}$ and $\mathbf{c}$ are defined differently from their definition for log-ratio PCA in the main text.) We then calculate the transformed matrix $\mathbf{S}$ as $s_{ij} = \frac{p_{ij} - r_i c_j}{\sqrt{r_i c_j}}$, which is then analyzed by SVD. The scores of the CA ordination diagram are derived from the left and right singular vectors by division by the square root of $\mathbf{r}$ and $\mathbf{c}$, respectively (Greenacre, 2007; ter Braak & Verdonschot, 1995) (To obtain a biplot, either the left or the right singular vector must be multiplied by the singular value as well (Greenacre, 2007).) For a recent account how CA and CCA relate to the RCM model with Poisson error (i.e. without overdispersion), see Appendix 3.4 of ter Braak (2019). In the diagnostics plots for CA we use $\mathbf{r}$ as defined for CA.

With CA and CCA we can either analyze the raw counts or transformed versions of the data (i.e. square root or log transformed). Given the over-dispersed nature of amplicon sequencing data, it is likely that when analyzing the raw counts one or a couple taxa/observations receive a disproportionate large influence in a CA fit. In such cases it may be better to analyze transformed data. By transforming the data we "shrink" the counts, reducing the influence of the high counts. Note that the weights in CA are based on the transformed data

(i.e. thus high abundant taxa receive less weight after transformation).

3.4 Anosim and Permanova

Two methods that are popular for analyzing microbiome data are anosim (Clarke, 1993) and permanova (Anderson, 2001). The default distance measures that is typically for these methods is bray-curtis (BC). Given that the BC distance is affected by the library size ((Greenacre, 2017)), we first transform the counts to proportions. We then calculate BC distances on the proportions. Note that this makes the BC distance equivalent to the Manhattan distance on the proportions (they differ by a factor of 2), and it also equivalent to Whittaker's index of association ((Whittaker, 1952)). As these methods are for testing, they are only included in the type 1 error rate and power simulations. These analyses were done with `R` package `vegan` ((Oksanen et al., 2019)).

3.5 RCM

RCM (Row-Column Model) fits a log-linear model with a negative binomial glm (Hawinkel et al., 2019). The log-linear model contains parameters for all rows and columns of $\mathbf{Y}$ and one or more bilinear interaction terms. RCM does require row centering (i.e. clr) and is expected to be insensitive to any influence of the library size via the row parameters. The sample and species scores per ordination axes are estimated sequentially. One advantage of methods based on a glm is that we model the expected value, which is strictly positive in a log-linear model, and thus a pseudo count is not needed. One issue with RCM (and model-based methods in general) is that underlying maximization problem that needs to be solved is complex, which may come at the price of reduced power and/or failed convergences.

In order to see the relation between RCM and log-ratio PCA, log-ratio PCA must be re-expressed as a linear model for $log(y_{ij})$ with free row and column parameters and bilinear interaction terms (Aitchison, 1983). This linear model is identical to the linear predictor of RCM. Thus, whereas log-ratio PCA models $log(y_{ij})$, RCM models $log(Ey_{ij})$.

Significance testing is not implemented in the RCM R package. For this supplement, we implemented significance testing by randomly permuting the treatment labels among the samples. The RCM R package includes its own filtering step which we disabled to make results comparable. Due to computational burden of the row-column model (RCM), we had to reduce its number of simulations. On the simulated data, RCM either converged after 3 iterations or did not converge after 2000 iterations. To speed up the fitting process we set the maximum number of iterations to 5. As a result many fits failed in both simulations and permutations. In both cases we only counted the converged fits. For each scenario we used 500 simulations of which 200-250 succeeded. The number of permutations was set to 200 of which 50-150 succeeded. After the additional filtering step to remove low abundance taxa, there were no failed convergence.

4 Results

4.1 log-ratio PCA

In the main text we show that log-ratio PCA is influenced by variation in $\mathbf{r}$. After additional filtering, log-ratio PCA identifies the effect of $\mathbf{x}$ on the 1st axis in all three examples (fig. 2). There are also no strong negative values for $\rho_{\mathbf{Sr}}$ for low abundance taxa (fig. 2). Also, there are neither V-shapes nor negative trends in the plots of log-contribution versus the log of the taxon mean. With zero imputation using geometric Bayesian multiplicative imputation (GBM), a similar result is obtained in the simulated and rice example In the biting midges example we still see negative values for $\rho_{\mathbf{Sr}}$ and high contributions amongst low taxa (fig. 3). Figure 3 displays the result from imputing the fractions. A similar result is obtained when imputing the counts, but due to the higher type 1 error when imputing counts (Table 1) we omitted this figure.

4.2 Weighted log-ratio PCA

Weighted log-ratio PCA identifies the effect of $\mathbf{x}$ on the 1st axis in all three examples and its correlation and contribution plots show positive trends against abundance (fig. 4). With the positive trends, the contribution plots show that weighted log-ratio PCA succeeded in decreasing the contributions of the low abundance taxa compared to those of the higher abundant taxa, suggesting that the effect of $\mathbf{r}$ is reduced compared to the unweighted analysis. However, the correlation plots shows even stronger negative correlations ($\rho_{\mathbf{Sr}}$) for low abundance taxa than unweighted LRA (figure 4 C, F, I), showing that the effect of $\mathbf{r}$ is still present in the transformed matrix $\mathbf{S}$. Additional filtering removes most of the strong negative correlations for $\rho_{\mathbf{Sr}}$ (Fig 5).

4.3 Log proportions PCA

When analyzing the simulated example as log proportions we see that large variation in $\mathbf{r}$ has little influence on the result and we can still identify the effect of $\mathbf{x}$ in both simulated and data examples (Figure 6). Filtering away low abundance taxa does not seem to have a great positive or negative effect on identifying $\mathbf{x}$ (Figure 7).

4.4 Correspondence analysis

Correspondence analyses with untransformed counts is strongly influenced by one outlying observation in each of the three examples and as such it poorly displays the variation present in the data (fig. 8). With the square root transformed counts (fig. 9), CA is able to identify the effect of $\mathbf{x}$ on the 1st axis for all examples, but for the rice example the 2nd axis detects an outlying observation. A log transformation removes the outliers from the first two axes, but appears to introduce some library size dependency as is visible in the simulation and biting midges examples where the effect of $\mathbf{x}$ is pushed from the first axis to the 2nd axis (fig. 10). Unfortunately, the diagnostics designed for log-ratio PCA do not clearly indicate the dependence; the correlation plots do not show

strong negative correlations for low abundance taxa and the contribution plots are hardly V-shaped.

After an additional filtering step to remove low abundance taxa we see improvements when analyzing the untransformed (fig. 11), square root (fig. 12) and log transformed counts (fig. 13). With untransformed and square root transformed data CA is less influenced by outliers. For the log transformed data, the effect of $\mathbf{x}$ is well identified on the 1st axis.

4.5 RCM

On the simulated data RCM is able to identify the effect of $\mathbf{x}$ without extra filtering. An additional filtering step has little influence on this result. With the biting midges example RCM is not able to identify the effect $\mathbf{x}$, with or without extra filtering (fig. 14).
With the rice example, RCM identifies the effect of $\mathbf{x}$ on the the 2nd axis (fig. 14). With additionally filtering RCM is able to identify the effect of $\mathbf{x}$ on the 1st axis (fig. 14). Note that the RCM default filtering removes 528 out of 650 taxa and also identifies the effect of $\mathbf{x}$ on the 1st axis (result not shown).

4.6 Summary

As indicated in Fig 2-14, a large number of zeroes with variability in library size can distort the ordination axes of an unconstrained analysis for a number of methods. Filtering the zeroes in most cases removes the distorting effect. In the power and type 1 simulation, the scenario where $\mathbf{x}$ and $\mathbf{r}$ are correlated and $\sigma_a = 1$ is particularly challenging for most methods (Table 1, and main document Fig 5). When analyzing such data, it can be useful to do an additional filtering step to reduce the number of zeroes. This improves the control of the type 1 error (Table 2, and main document Fig 6). Given the relationship between filtering and type 1 error, most of the evaluated methods do not have a guaranteed control of type 1 error in this situation. It is reassuring is that without a correlation between $\mathbf{x}$ and $\mathbf{r}$, type 1 error is under control for all values of σ_a. If $\mathbf{x}$ and $\mathbf{r}$ are uncorrelated, there are a number methods that have a good power irrespectively of the value of σ_a, e.g. log proportions RDA, log-ratio RDA with GBM imputation, and CA on square root transformed data. Without a correlation between $\mathbf{x}$ and $\mathbf{r}$ and for low values of σ_a, log-ratio RDA is the most powerful method. The performance of CCA is dependent on the applied transformation (and by extension thus on the distribution of the data).

5 Additional data examples

To further illustrate the row centering problem of log-ratio PCA, we investigated some publicly available well-known examples. For these examples we provide correlation and contribution plots, i.e. the plots of contributions and $\rho_{\mathbf{Sr}}$ versus the log mean abundance per taxon. We examine these examples both after a mild filtering step, and after a more stringent filtering step.

5.1 Human gut

The human gut data originally come from Yatsunenko et al. (2012) and were, amongst others, used by Jonsson et al. (2019), and were downloaded from the supplementary material accompanying the latter. In the mild filtering step we removed taxa that were absent in 10 (out of 110) or more samples. In the stringent filtering step we only kept taxa that occurred at least 50 times and had a abundance of minimally 2000 reads. The library size with these data varies from 4672 to 173464. The data contains 4219 taxa with 28% zeroes after the first filtering step, and 976 taxa with close to 0% zeroes after the more stringent filtering step.

If we apply log-ratio PCA to these data after mild filtering (fig. 15) we see strong negative values for $\rho_{\mathbf{Sr}}$ for the low abundance taxa. We also see that the 2nd axis has relatively high contributions amongst the low abundance taxa; the pattern shows a (filled) V-shape. Both features are indicative of the influence of $\mathbf{r}$ and a problem with row centering. After the stringent filtering step there are no evident signals for problems with row centering, i.e. the correlations are centered around zero, and there is no pattern in the contributions of either the 1st or 2nd axis.

5.2 Lung data

The lung data set is one of the examples provided with the R package `metagenomeSeq` and come from Charlson et al. (2011). In the mild filtering step we removed taxa that were absent in 10 (out of 66) or more samples; the mildly filtered data contained 1029 taxa with 75% zeroes. After the stringent filtering step (minimal occurrence of 25, and minimal taxon abundance of 500 reads), these data contain 33 taxa with 39% zeroes. The library size with these data varies from 45 to 32446.

If we apply log-ratio PCA to these data after the mild filtering step, we see the patterns associated with the problem of row centering (fig. 16): strong negative correlations for $\rho_{\mathbf{Sr}}$ for the low abundance taxa and a (filled) V-shape in the contribution plot. After the stringent filtering most of these diagnostic features are gone, but there seem to be still some low abundance taxa with negative values for $\rho_{\mathbf{Sr}}$, likely caused by the 39% remaining zeroes. It seems that in this example we are not able to bring the number of zeroes sufficiently down despite removing the majority of the taxa.

5.3 Global patterns

The global patterns data set is one of the examples provided with the R package `phyloseq` and come from Caporaso et al. (2011). In the mild filtering step we removed taxa that were absent in 5 (out of 26) or more samples, so retaining 8367 taxa with 63% zeroes. After the stringent filtering step (minimal occurrence of 20, and minimal taxon abundance of 1000 reads), the data contains 510 taxa with 11% zeroes. The library size with these data varies from 55636 to 2341204.

If we apply log-ratio PCA to these data after the mild filtering step, we see the diagnostic features associated with the problem of row centering (fig. 17). We see strong negative values for $\rho_{\mathbf{Sr}}$, with additionally some dichotomous effect of unclear origin. We also see a relatively high contribution, and the V-shape is visible, amongst the low abundance taxa.

After an extra filtering step there are no evident signals for problems with row centering. The correlations $\rho_{\mathbf{Sr}}$ are centered around zero without obvious pattern and the V-shape in the contribution plot has disappeared.

References

Aitchison, J (1983), Principal component analysis of compositional data, *Biometrika*, 70, https://doi.org/10.2307/2335943.

Anderson, M (2001), A new method for non-parametric multivariate analysis of variance, *Austral Ecology*, 26(1), 32-46, https://doi.org/10.1111/j.1442-9993.2001.01070.pp.x.

Caporaso, J, Lauber, C, Walters, W, Berg-Lyons, D, Lozupone, C, Turnbaugh, P, Fierer, N & Knight, R (2011), Global patterns of 16s rrna diversity at a depth of millions of sequences per sample, *Proceedings of the National Academy of Sciences*, 108(Supplement 1), 4516-4522, https://doi.org/10.1073/pnas.1000080107.

Charlson, E, Bittinger, K, Haas, A, Fitzgerald A.S., I, Frank, Yadav, A, Bushman, F & Collman, R (2011), Topographical continuity of bacterial populations in the healthy human respiratory tract, *American journal of respiratory and critical care medicine*, 184(8), 957-963, https://doi.org/10.1164/rccm.201104-0655OC.

Clarke, K (1993), Non-parametric multivariate analyses of changes in community structure, *Australian Journal of Ecology*, 18(1), 117-143, https://doi.org/10.1111/j.1442-9993.1993.tb00438.x.

Gloor, G, Macklaim, J, Pawlowsky-Glahn, V & Egozcue, J (2017), Microbiome datasets are compositional: And this is not optional, *Frontiers in Microbiology*, 8, https://doi.org/10.3389/fmicb.2017.02224.

Greenacre, M (2007), *Correspondence Analysis in Practice*, Chapman and Hall/CRC.

Greenacre, M (2017), Size and shape in the measurement of multivariate proximity, *Methods in Ecology and Evolution*, 8, 1415-1424, https://doi.org/0.1111/2041-210X.12776.

Greenacre, M (2018), *Compositional Data Analysis in Practice*, Chapman and Hall/CRC.

Greenacre, M & Lewi, P (2009), Distributional equivalence and subcompositional coherence in the analysis of compositional data, contingency tables and ratio-scale measurements, *Journal of Classification*, 26(1), 29-54, https://doi.org/10.1007/s00357-009-9027-y.

Hawinkel, S, Kerckhof, F, Bijnens, L & Thas, O (2019), A unified framework for unconstrained and constrained ordination of microbiome read count data, *PLoS ONE*, 14(2), 1-20, https://doi.org/10.1371/journal.pone.0205474.

Jonsson, V, Österlund, T, Nerman, O & E., K (2019), Modelling of zero-inflation improves inference of metagenomic gene count data, *Statistical Methods in Medical Research*, 28, https://doi.org/doi.org/10.1177/0962280218811354.

Martín-Fernández, J, Hron, K, Templ, M, Filzmoser, P & J., PA (2015), Bayesian-multiplicative treatment of count zeros in compositional data sets, *Statistical Modelling*, 15, https://doi.org/10.1177/1471082X14535524.

McMurdie, P & Holmes, S (2014), Waste not, want not: Why rarefying microbiome data is inadmissible, *PLOS Computational Biology*, 4, https://doi.org/10.1371/journal.pcbi.1003531.

Oksanen, J, Kindt, R, Legendre, P, OH́ara, B, Simpson, G, Solymos, P, Henry, M, Stevens, H & Wagner, H (2019), The vegan package, *Community ecology package*.

Palarea-Albaladejo, J & Martín-Fernández, J (2015), zcompositions – r package for multivariate imputation of left-censored data under a compositional approach, *Chemometrics and Intelligent Laboratory Systems*, 143, 85-96, https://doi.org/10.1016/j.chemolab.2015.02.019.

Rivera-Pinto, J, Egozcue, J, Pawlowsky-Glahn, V, Paredes, R, Noguera-Julian, M & Calle, M (2018), Balances: a new perspective for microbiome analysis, *mSystems*, 3, https://doi.org/10.1128/mSystems.00053-18.

ter Braak, C (2019), New robust weighted averaging- and model-based methods for assessing trait-environment relationships, *Methods in Ecology and Evolution*, 10(11), 1962-1971, https://doi.org/10.1111/2041-210X.13278.

ter Braak, C & Verdonschot, P (1995), Canonical correspondence analysis and related multivariate methods in aquatic ecology, *Aquatic Sciences*, 57(3), 255-289, https://doi.org/10.1007/BF00877430.

Whittaker, R (1952), A study of summer foliage insect communities in the great smoky mountains, *Ecological Monographs*, 22(1), 1-44, https://doi.org/10.2307/1948527.

Yatsunenko, T, Rey, F, Manary, M, Trehan, I, Dominguez-Bello, M, Contreras, M, Magris, M, Hidalgo, G, Baldassano, R, Anokhin, A, Heath, A, Warner, B, Reeder, J, Kuczynski, J, Caporaso, J, Lozupone, C, Lauber, C, Clemente, J, Knights, D, Knight, R & Gordon, J (2012), Human gut microbiome viewed across age and geography, *Nature*, 14, https://doi.org/10.1038/nature11053.

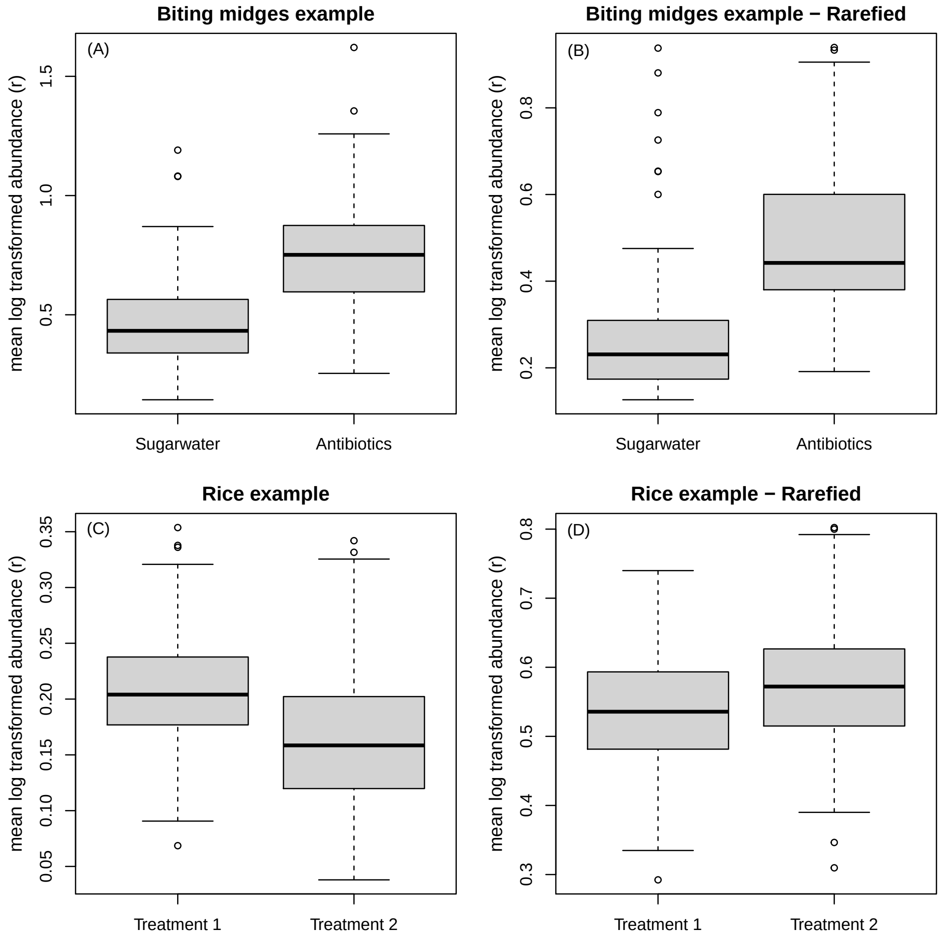

Figure 1: The row mean of the log transformed abundance data (i.e. $\mathbf{r}$) for both example data sets, without (A, C) and with (B, D) rarefaction, versus the applied treatment. Rarefaction depth was set to the lowest library size. The figures for the rarefied data show that $\mathbf{r}$ can vary even when (rarefied) library sizes are equal.

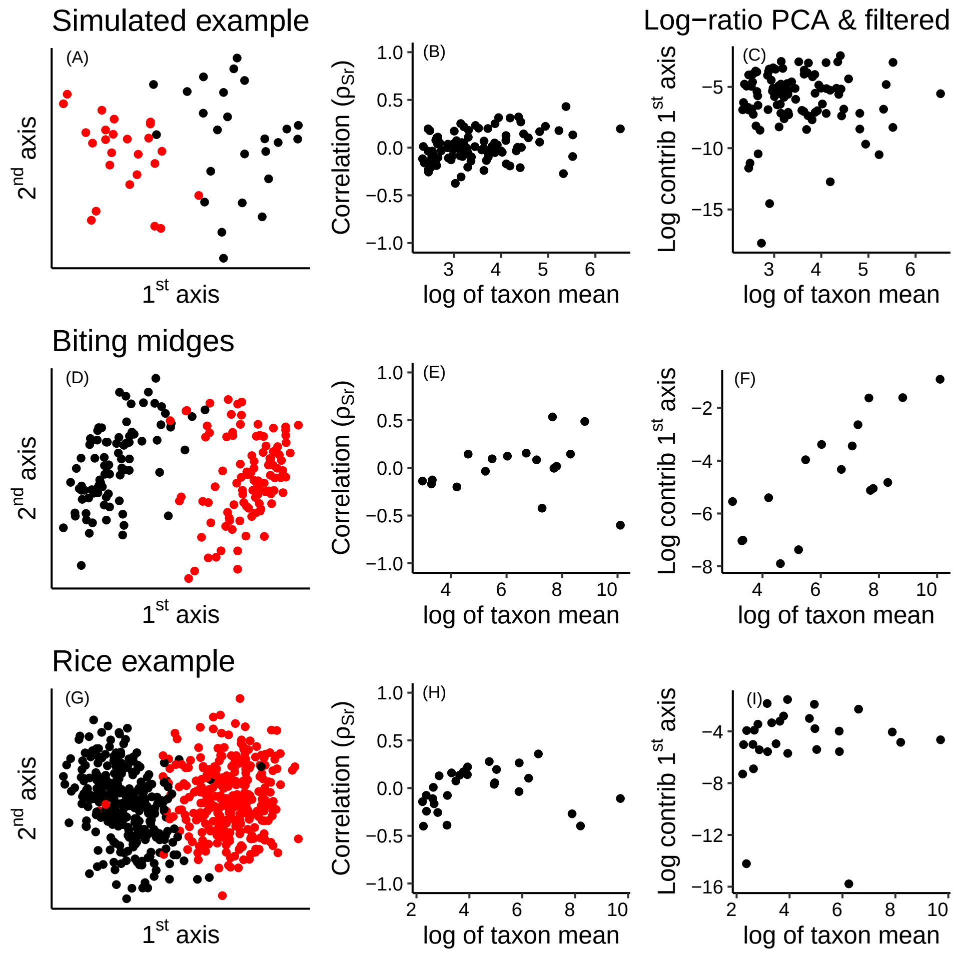

Figure 2: Results for log-ratio PCA after additional filtering for the simulated example (A-C), the biting midges example (D-F), and the rice example (G-I). From left to right the figures display the 1st and 2nd principal axis where the colors indicate the treatment groups, correlation ($\rho_{\mathbf{Sr}}$) per taxon plotted against taxon abundance, and log contribution also plotted against taxon abundance.

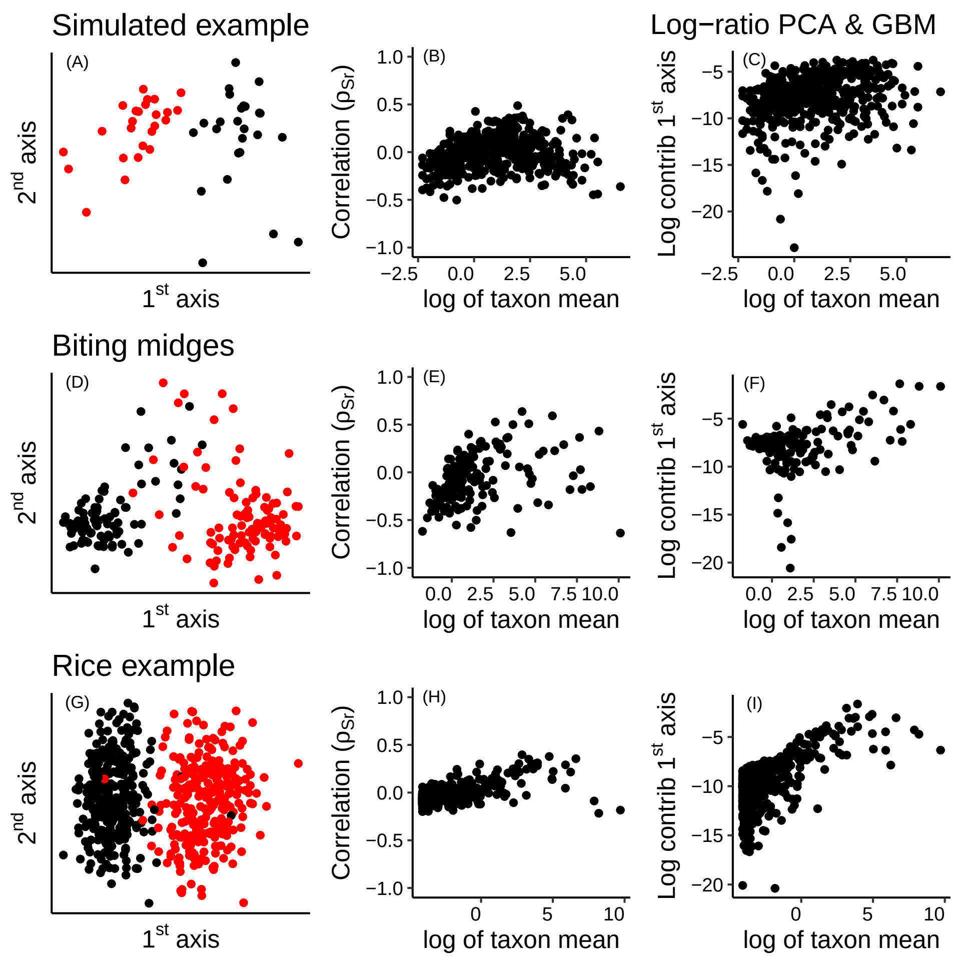

Figure 3: Results for log-ratio PCA after geometric Bayesian zero imputation (GBM) and additional filtering for the simulated example (A-C), the biting midges example (D-F), and the rice example (G-I). From left to right the figures display the 1st and 2nd principal axis where the colors indicate the treatment groups, correlation ($\rho_{\mathbf{Sr}}$) per taxon plotted against taxon abundance, and log contribution also plotted against taxon abundance.

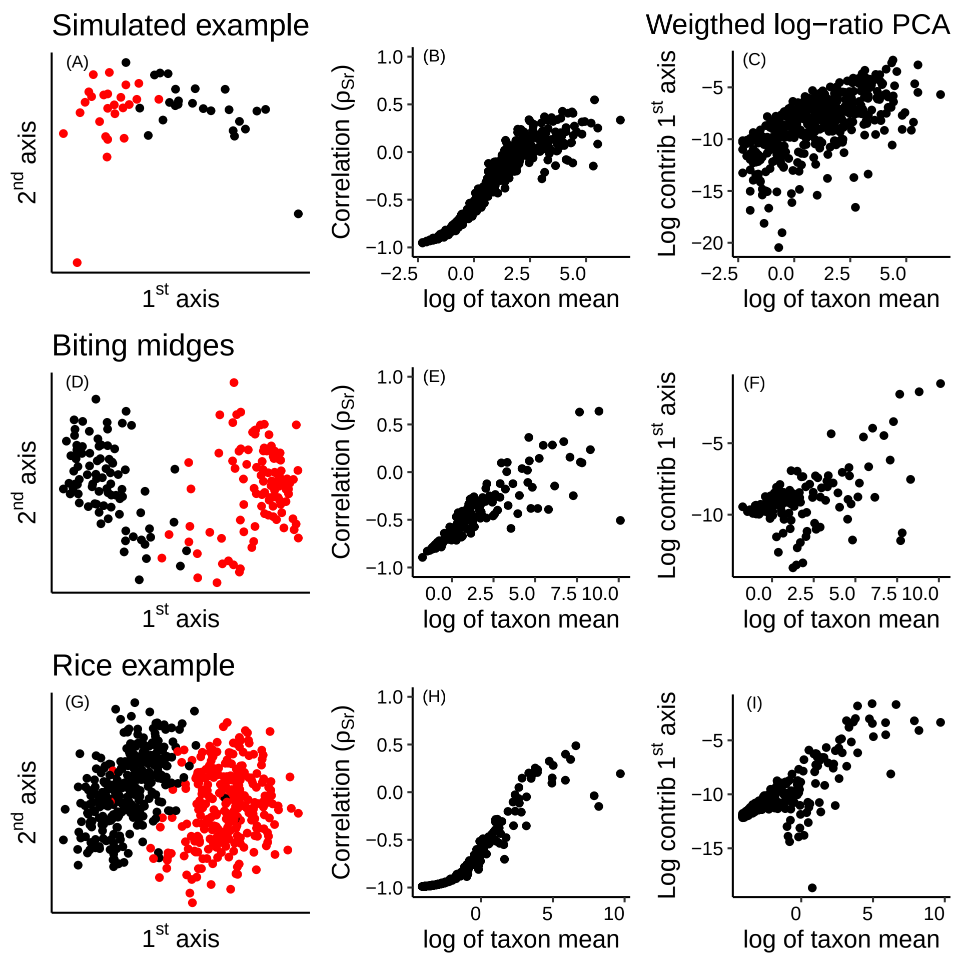

Figure 4: Results weighted log-ratio PCA for the simulated example (A-C), the biting midges example (D-F), and the rice example (G-I). From left to right the figures display the 1st and 2nd principal axis where the colors indicate the treatment groups, correlation ($\rho_{\mathbf{Sr}}$) per taxon plotted against taxon abundance, and log contribution also plotted against taxon abundance.

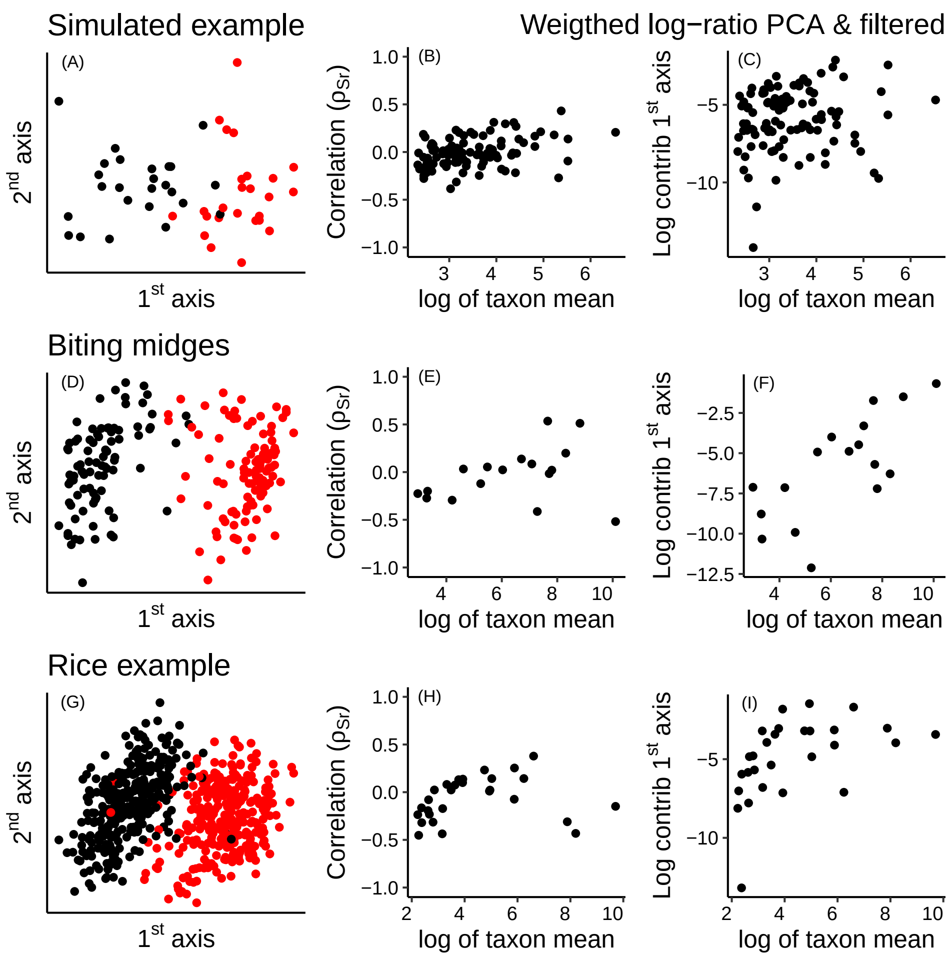

Figure 5: Results for weighted log-ratio PCA after extra filtering for the simulated example (A-C), the biting midges example (D-F), and the rice example (G-I). From left to right the figures display the 1st and 2nd principal axis where the colors indicate the treatment groups, correlation ($\rho_{\mathbf{Sr}}$) per taxon plotted against taxon abundance, and log contribution also plotted against taxon abundance.

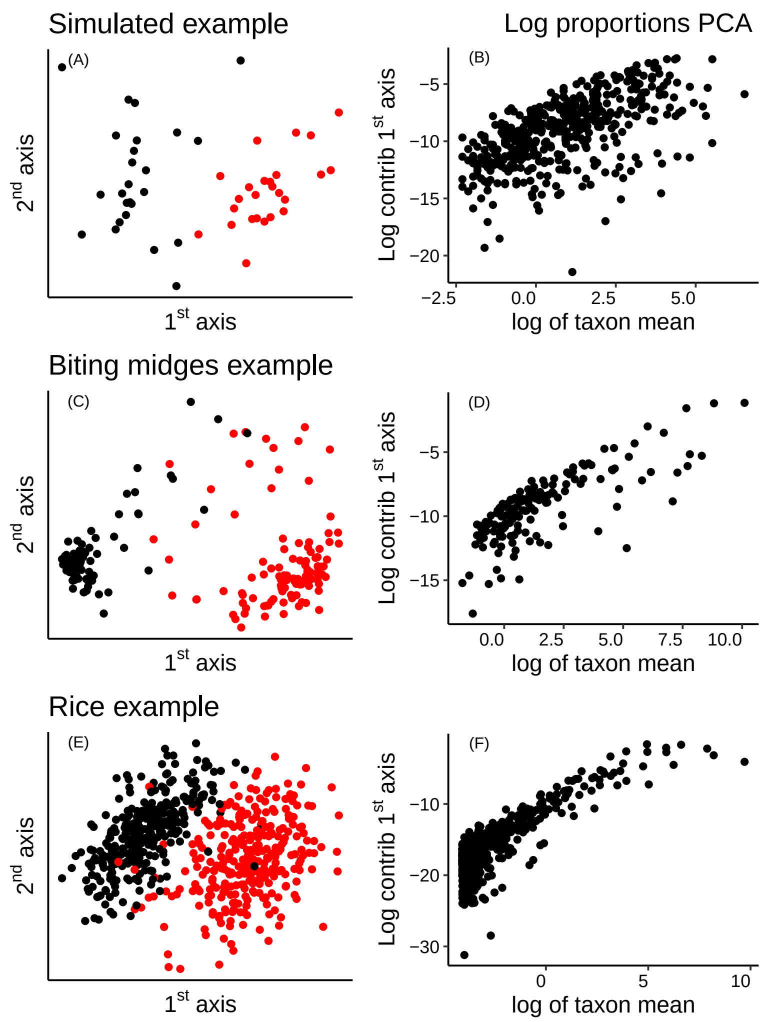

Figure 6: Results when analyzing the data as log proportions for the simulated example (A-B), the biting midges example (C-D), and the rice example (E-F). The left figures display the 1st and 2nd principal axis where the colors indicate the treatment groups, the right figures display the log contribution per taxon plotted against taxon abundance.

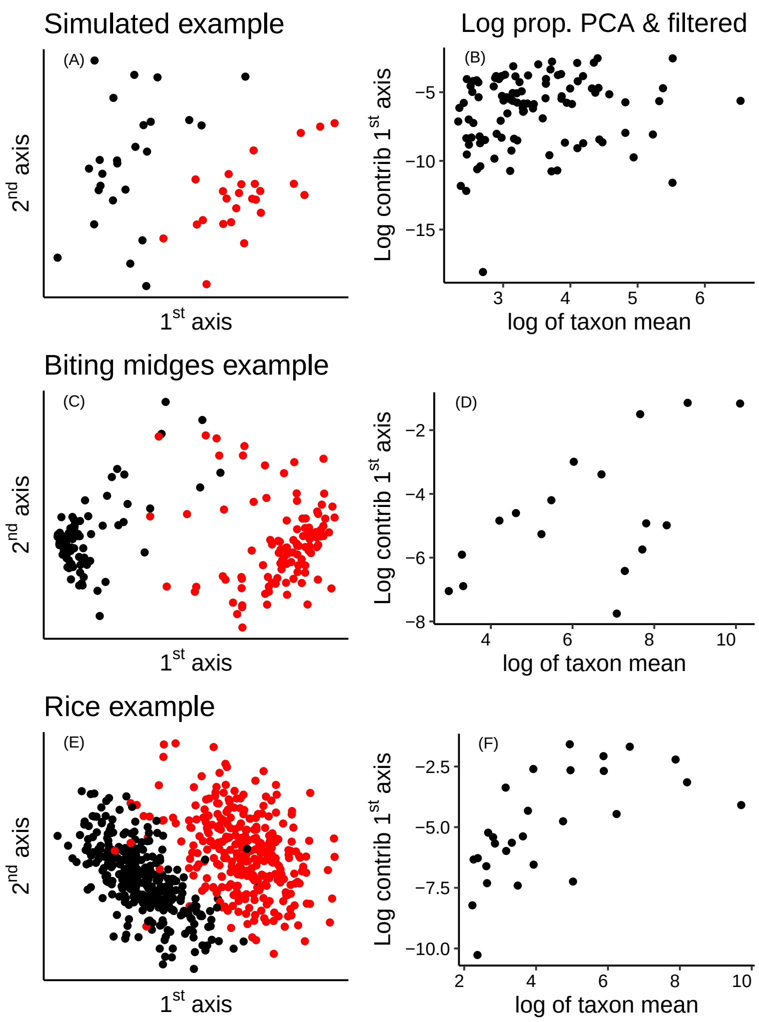

Figure 7: Results when analyzing the data as log proportions after additional filtering for the simulated example (A-B), the biting midges example (C-D), and the rice example (E-F). The left figures display the 1st and 2nd principal axis where the colors indicate the treatment groups, the right figures display the log contribution per taxon plotted against taxon abundance.

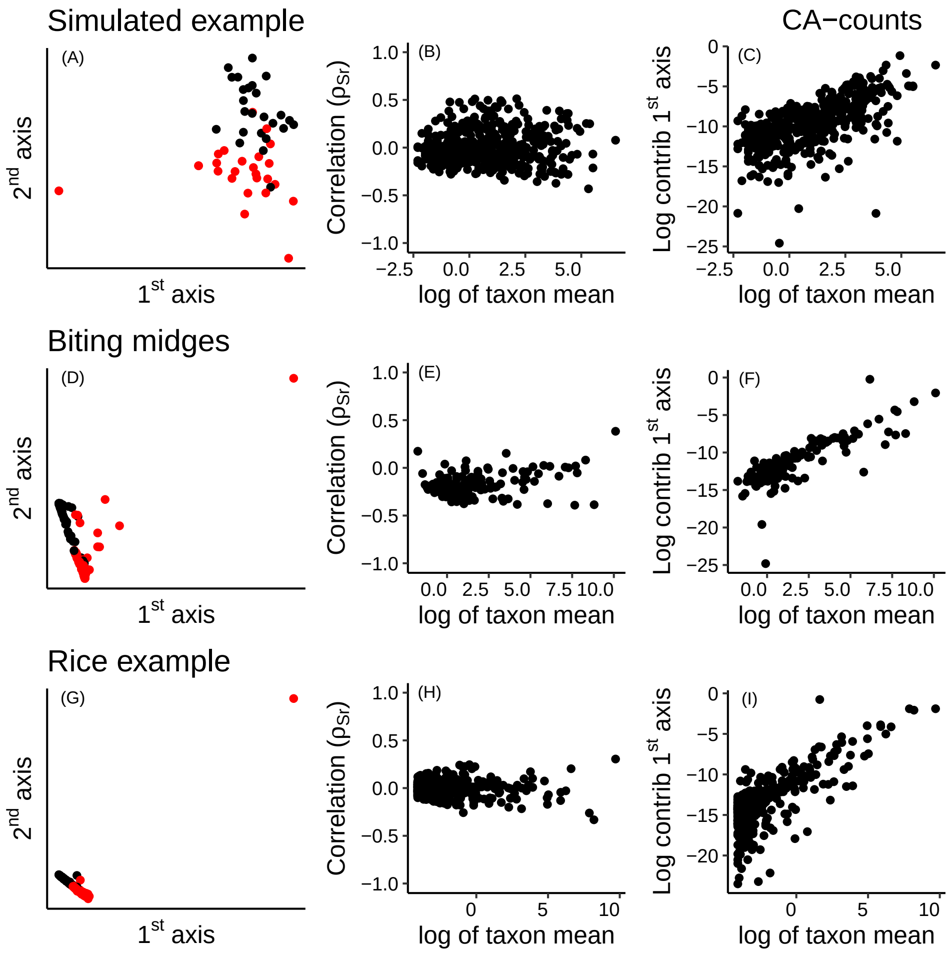

Figure 8: Results for CA when analyzing the raw counts for the simulated example (A-C), the biting midges example (D-F), and the rice example (G-I). From left to right the figures display the 1st and 2nd principal axis where the colors indicate the treatment groups, correlation ($\rho_{\mathbf{Sr}}$) per taxon plotted against taxon abundance, and log contribution also plotted against taxon abundance.

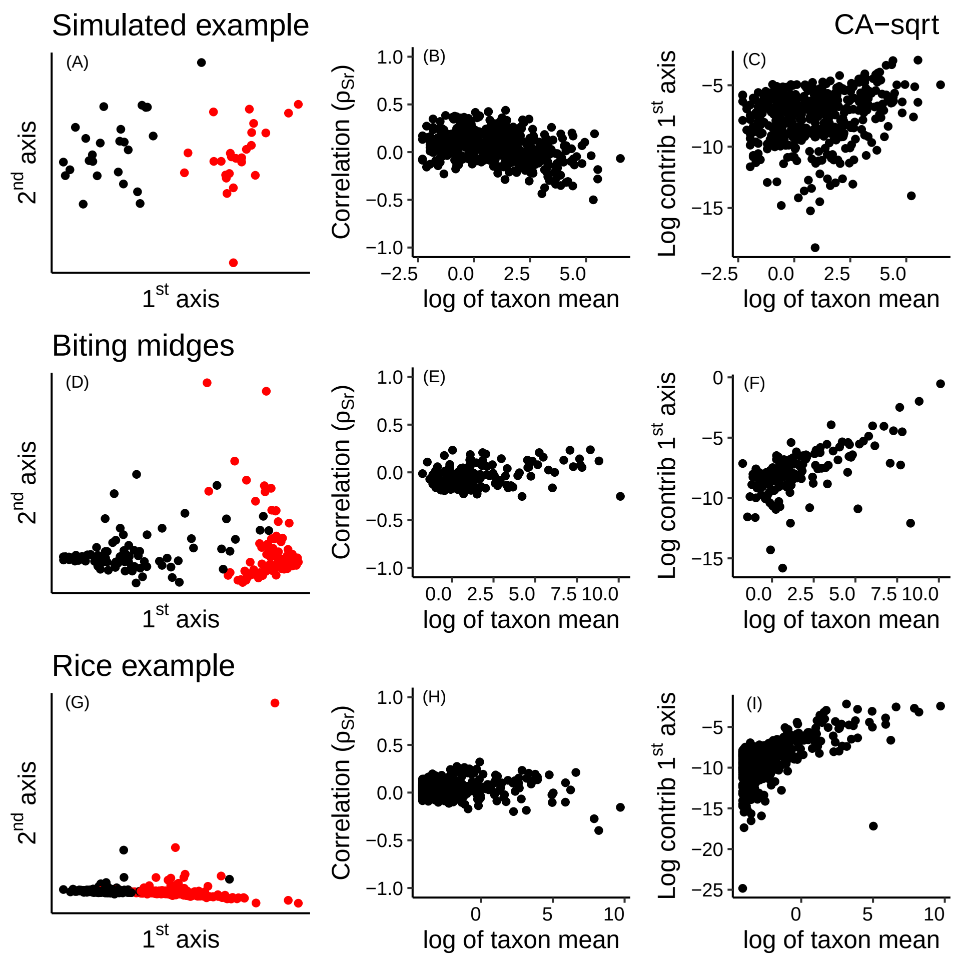

Figure 9: Results for CA after square root transforming the counts for the simulated example (A-C), the biting midges example (D-F), and the rice example (G-I). From left to right the figures display the 1st and 2nd principal axis where the colors indicate the treatment groups, correlation ($\rho_{\mathbf{Sr}}$) per taxon plotted against taxon abundance, and log contribution also plotted against taxon abundance.

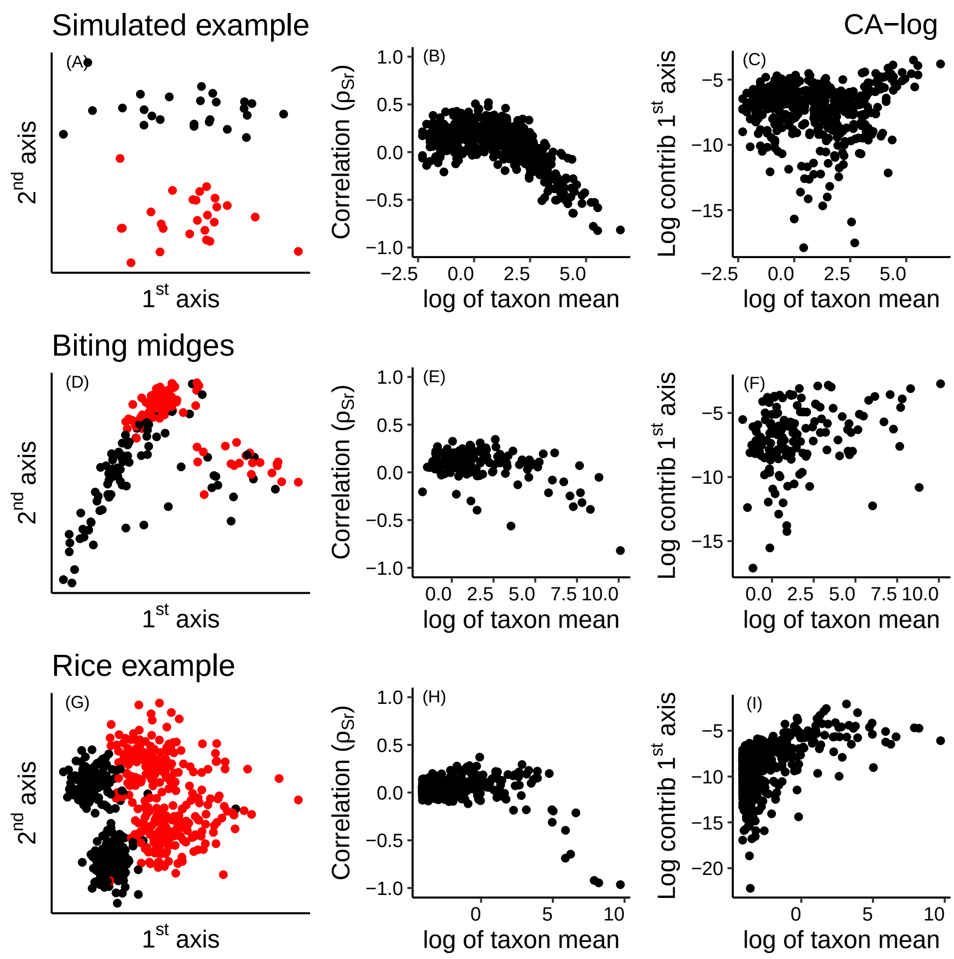

Figure 10: Results for CA after log transforming the counts for the simulated example (A-C), the biting midges example (D-F), and the rice example (G-I). From left to right the figures display the 1st and 2nd principal axis where the colors indicate the treatment groups, correlation ($\rho_{\mathbf{Sr}}$) per taxon plotted against taxon abundance, and log contribution also plotted against taxon abundance.

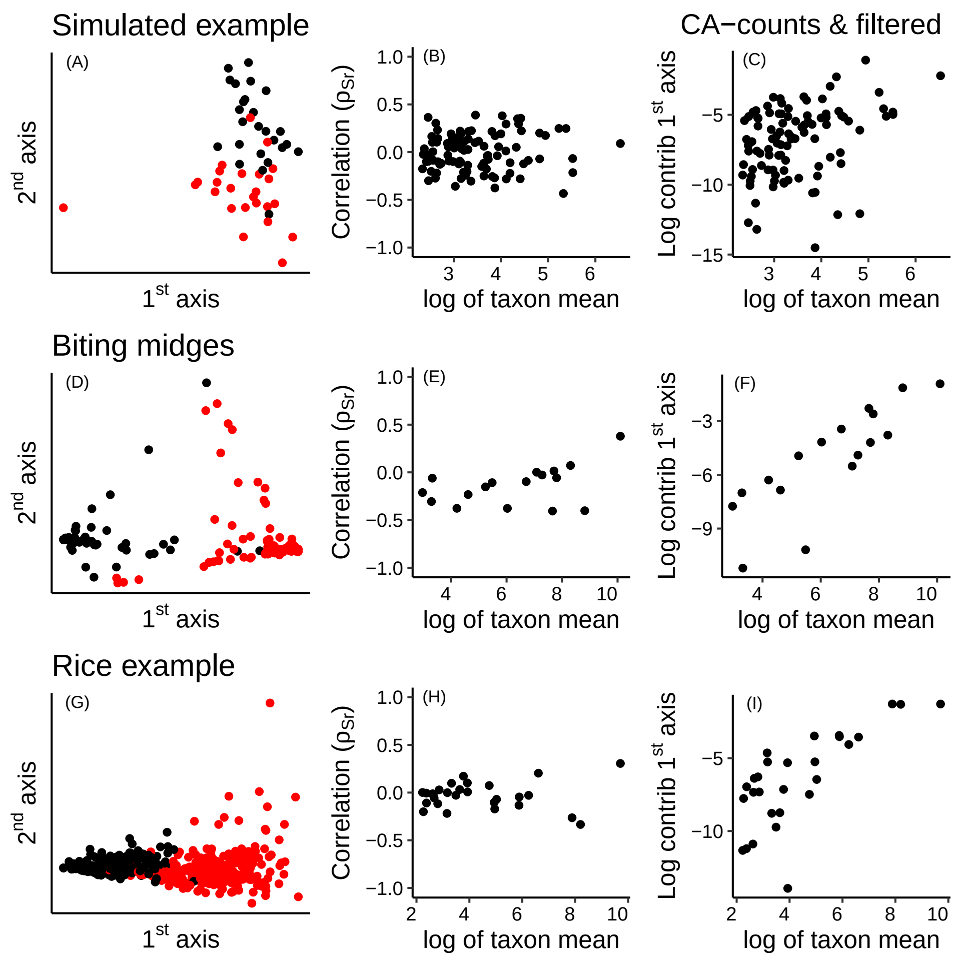

Figure 11: Results for CA when analyzing the raw counts after additional filtering for the simulated example (A-C), the biting midges example (D-F), and the rice example (G-I). From left to right the figures display the 1st and 2nd principal axis where the colors indicate the treatment groups, correlation ($\rho_{\mathbf{Sr}}$) per taxon plotted against taxon abundance, and log contribution also plotted against taxon abundance.

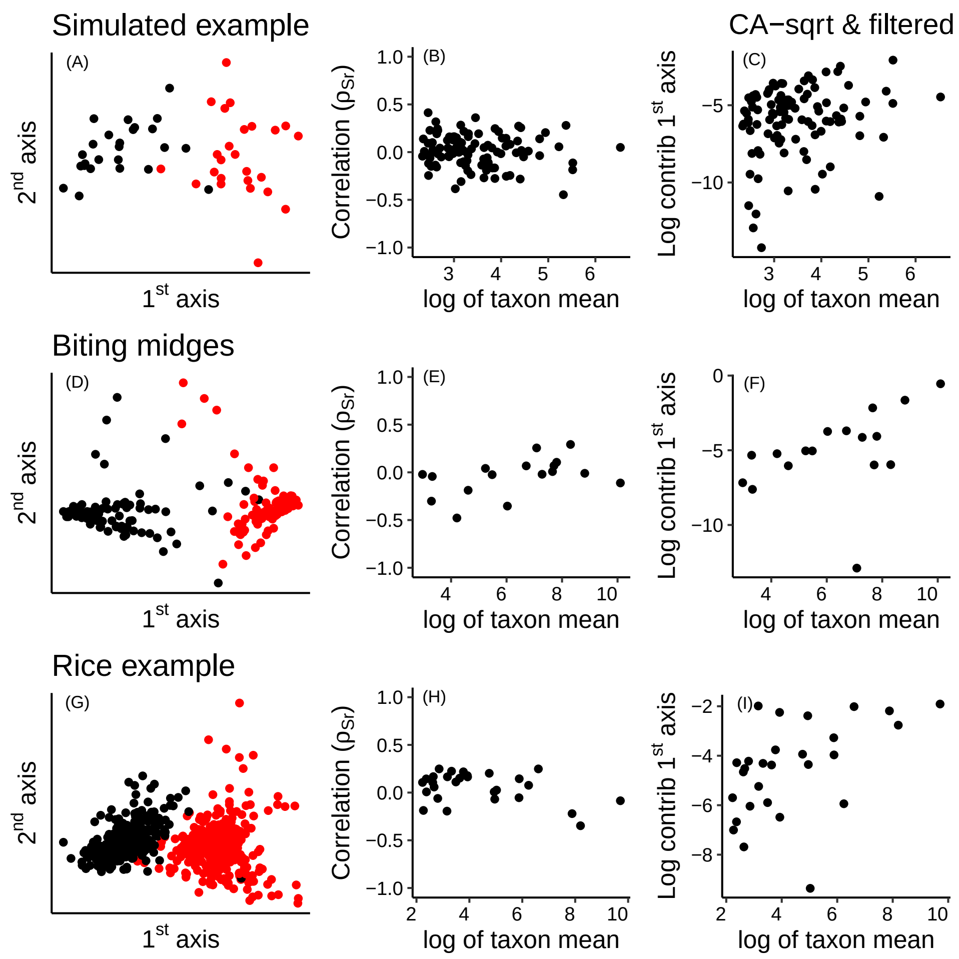

Figure 12: Results for CA after square root transforming the counts and additional filtering for the simulated example (A-C), the biting midges example (D-F), and the rice example (G-I). From left to right the figures display the 1st and 2nd principal axis where the colors indicate the treatment groups, correlation ($\rho_{\mathbf{Sr}}$) per taxon plotted against taxon abundance, and log contribution also plotted against taxon abundance.

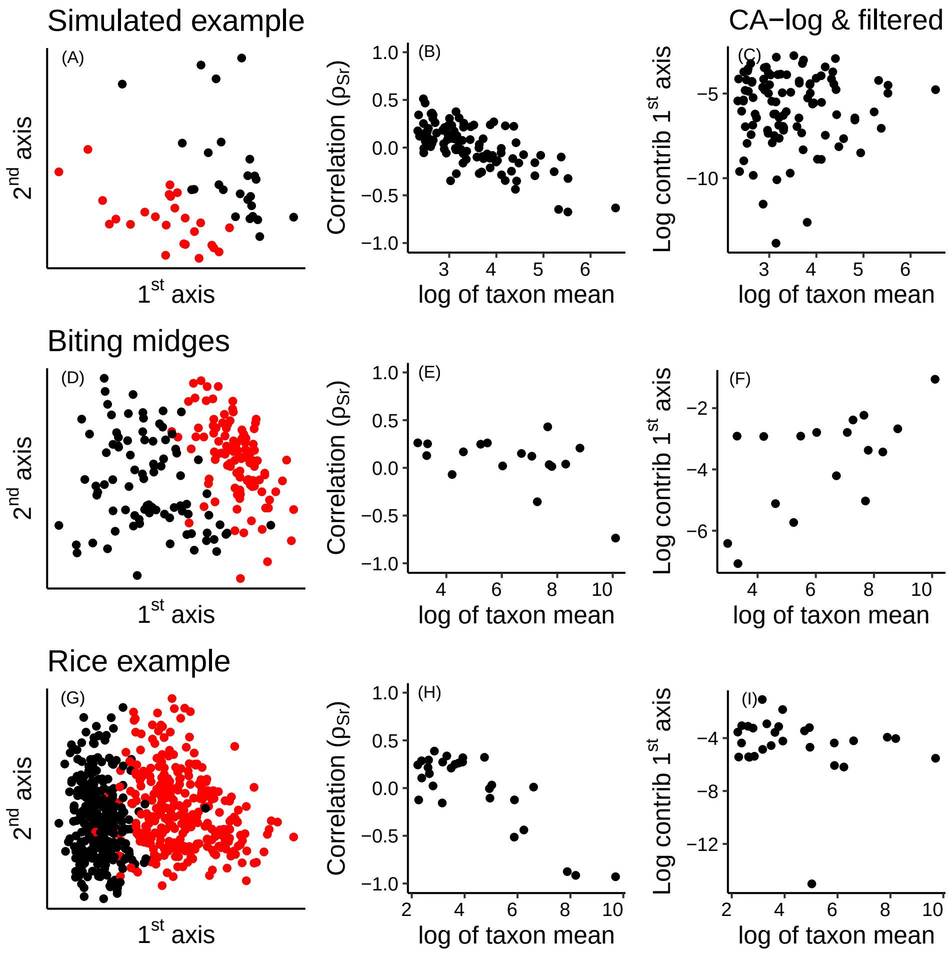

Figure 13: Results for CA after log transforming the counts after additional filtering for: the simulated example (A-C), the biting midges example (D-F), and the rice example (G-I). From left to right the figures display the 1st and 2nd principal axis where the colors indicate the treatment groups, correlation ($\rho_{\mathbf{Sr}}$) per taxon plotted against taxon abundance, and log contribution also plotted against taxon abundance.

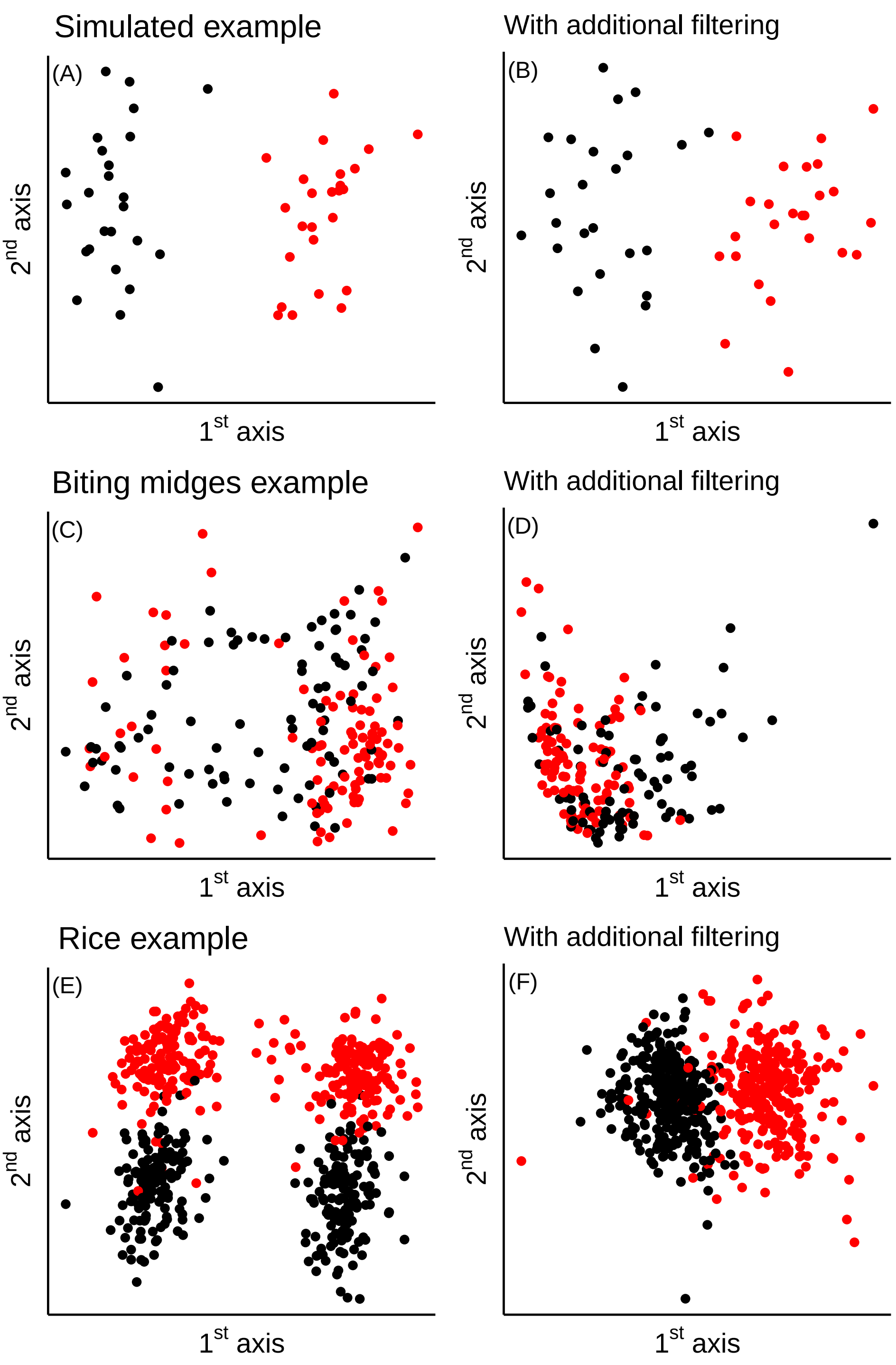

Figure 14: Results with RCM with and without additional filtering for the simulated example (A-B), the biting midges example (C-D), and the rice example (E-F). All figures display the 1st and 2nd principal axis where the colors indicate the treatment groups

σ_a	Method	Type 1 error[a]	Power[b]	Type 1 error with $\rho_{\mathbf{xr}}$[c]
0.25	Log-ratio RDA	0.05	0.80	0.09
	Log-ratio RDA GBM - counts	0.04	0.78	0.05
	Log-ratio RDA GBM - prop	0.05	0.79	0.04
	Weighted log-ratio RDA	0.04	0.57	0.05
	Log proportions RDA	0.05	0.69	0.05
	CCA - counts	0.04	0.35	0.05
	CCA - sqrt	0.04	0.78	0.03
	CCA - log	0.04	0.73	0.05
	Anosim (BC-prop)	0.05	0.29	0.05
	Permanova (BC-prop)	0.05	0.30	0.05
	RCM	0.004	0.34	0.008
0.5	Log-ratio RDA	0.05	0.68	0.67
	Log-ratio RDA GBM - counts	0.05	0.74	0.16
	Log-ratio RDA GBM - prop	0.05	0.76	0.05
	Weighted log-ratio RDA	0.05	0.53	0.13
	Log proportions RDA	0.05	0.68	0.04
	CCA - counts	0.06	0.30	0.03
	CCA - sqrt	0.04	0.75	0.06
	CCA - log	0.04	0.67	0.35
	Anosim (BC-prop)	0.05	0.29	0.05
	Permanova (BC-prop)	0.05	0.30	0.05
	RCM	0.008	0.39	0
1	Log-ratio RDA	0.05	0.17	0.99
	Log-ratio RDA GBM - counts	0.05	0.61	0.91
	log-ratio RDA GBM - prop	0.05	0.66	0.24
	Weighted log-ratio RDA	0.05	0.46	0.74
	Log proportions RDA	0.05	0.64	0.14
	CCA - counts	0.05	0.19	0.01
	CCA - sqrt	0.04	0.69	0.20
	CCA - log	0.04	0.31	0.93
	Anosim (BC-prop)	0.05	0.30	0.13
	Permanova (BC-prop)	0.05	0.29	0.11
	RCM	0.009	0.28	-[d]

Table 1: Type 1 and power for the various methods for three levels of row variation (σ_a); [a] indicates the type 1 error without correlation between $\mathbf{x}$ and $\mathbf{r}$ ($\gamma = 0$); [b] indicates the power (Fold change = 1.5) without correlation between $\mathbf{x}$ and $\mathbf{r}$ ($\gamma = 0$); [c] indicates the type 1 error when there is a correlation between $\mathbf{x}$ and $\mathbf{r}$ ($\gamma = 2$). For all methods expect RCM the type 1 error and power were determined by counting the number of p-values below 0.05 across 2000 simulations. For RCM we did between 200-250 simulations, expect for cell [d] where most simulations failed.

σ_a	Method	Type 1 error[a]	Power[b]	Type 1 error with $\rho_{\mathbf{xr}}$[c]
0.25	Log-ratio RDA	0.05	0.40	0.05
	Log-ratio RDA GBM - counts	0.05	0.39	0.05
	Log-ratio RDA GBM - prop	0.05	0.38	0.05
	Weighted log-ratio RDA	0.04	0.36	0.05
	Log proportions RDA	0.05	0.41	0.05
	CCA - counts	0.04	0.27	0.05
	CCA - sqrt	0.04	0.41	0.04
	CCA - log	0.04	0.37	0.04
	Anosim (BC-prop)	0.05	0.24	0.05
	Permanova (BC-prop)	0.05	0.24	0.05
	RCM	0.02	0.24	0.01
0.5	Log-ratio RDA	0.05	0.42	0.05
	Log-ratio RDA GBM - counts	0.04	0.37	0.04
	Log-ratio RDA GBM - prop	0.05	0.38	0.05
	Weighted log-ratio RDA	0.06	0.36	0.04
	Log proportions RDA	0.05	0.41	0.04
	CCA - counts	0.06	0.24	0.03
	CCA - sqrt	0.05	0.41	0.03
	CCA - log	0.03	0.37	0.09
	Anosim (BC-prop)	0.05	0.24	0.04
	Permanova (BC-prop)	0.05	0.24	0.04
	RCM	0.01	0.19	0.01
1	Log-ratio RDA	0.05	0.47	0.09
	Log-ratio RDA GBM - counts	0.05	0.41	0.08
	Log-ratio RDA GBM - prop	0.05	0.40	0.08
	Weighted log-ratio RDA	0.05	0.38	0.02
	Log proportions RDA	0.06	0.47	0.09
	CCA - counts	0.05	0.17	0.01
	CCA - sqrt	0.04	0.40	0.01
	CCA - log	0.04	0.38	0.77
	Anosim (BC-prop)	0.05	0.24	0.06
	Permanova (BC-prop)	0.05	0.26	0.05
	RCM	0.002	0.10	0.01

Table 2: Type 1 and power after additional filtering for the various methods and three levels of row variation (σ_a); [a] indicates the type 1 error without correlation between $\mathbf{x}$ and $\mathbf{r}$ ($\gamma = 0$); [b] indicates the power (Fold change = 1.5) without correlation between $\mathbf{x}$ and $\mathbf{r}$ ($\gamma = 0$); [c] indicates the type 1 error when there is a correlation between $\mathbf{x}$ and $\mathbf{r}$ ($\gamma = 2$). The type 1 error and power were determined by counting the number of p-values below 0.05 across 2000 simulations.

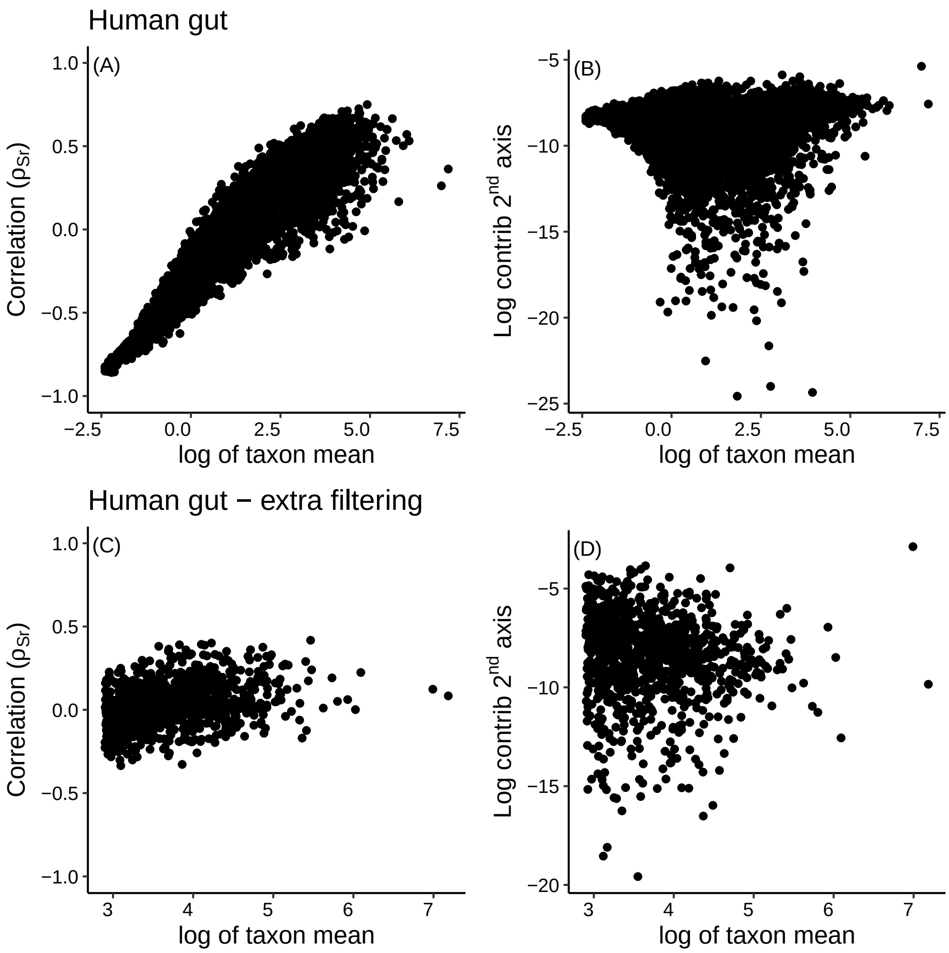

Figure 15: Correlation ($\rho_{\mathbf{xr}}$) (A, C) and contribution (B, D) versus log mean taxon abundance for the human gut data set. Top figures are with mild filtering of low abundance taxa, and bottom figure with a more stringent filtering step.

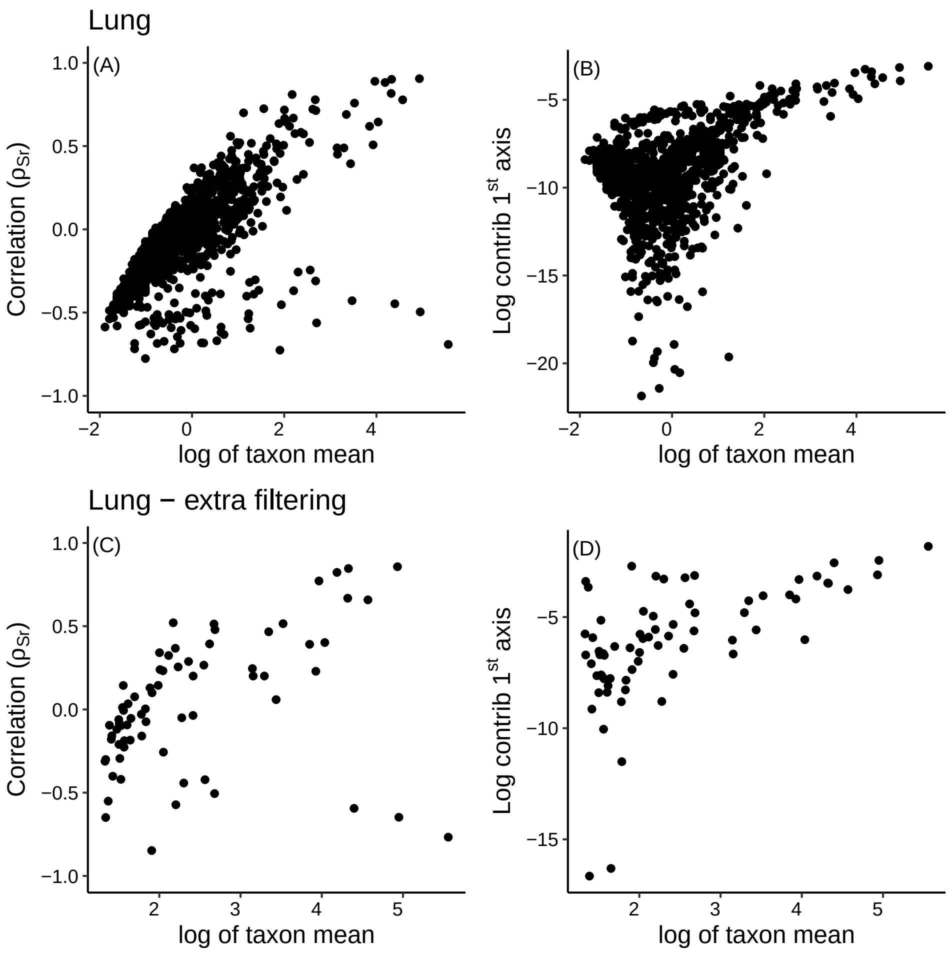

Figure 16: Correlations ($\rho_{\mathbf{xr}}$) (A, C) and contributions (B, D) versus log mean taxon abundance for the lung data set. Top figures are with mild filtering of low abundance taxa, and bottom figure with a more stringent filtering step.

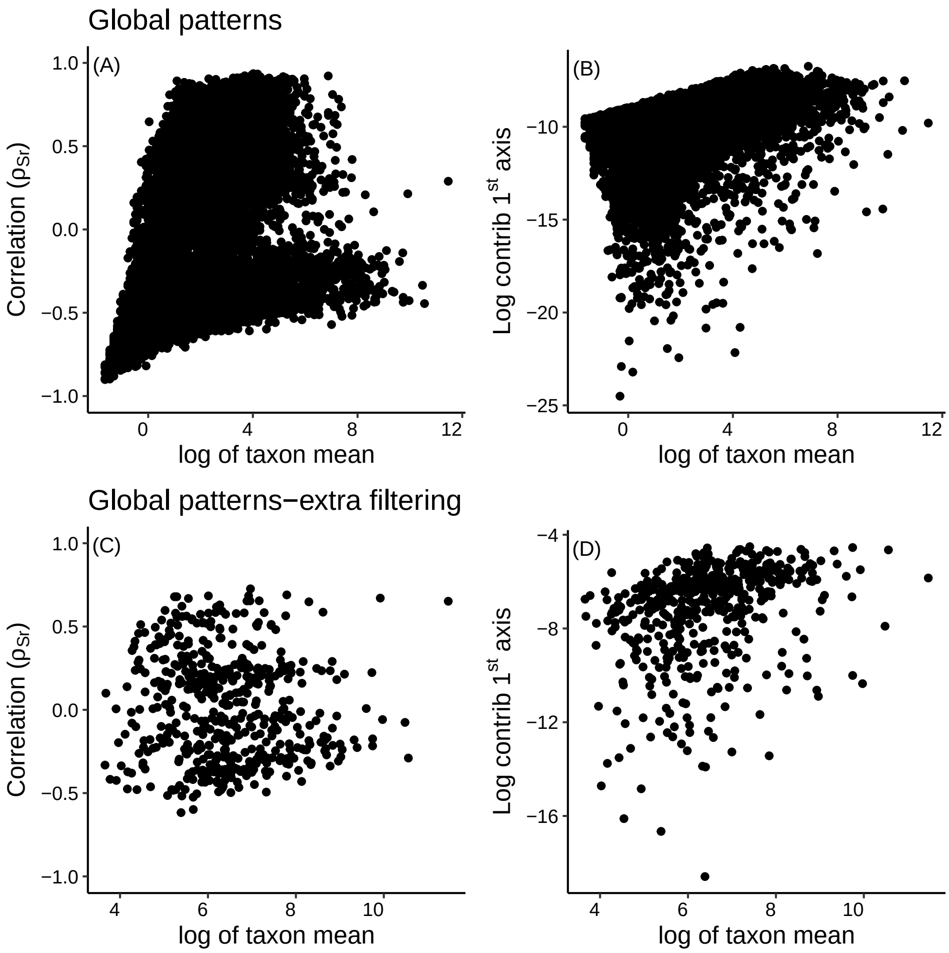

Figure 17: Correlations ($\rho_{\mathbf{xr}}$) (A, C) and contributions (B, D) versus log mean taxon abundance for the global patterns data set. Top figures are with mild filtering of low abundance taxa, and bottom figure with a more stringent filtering step.

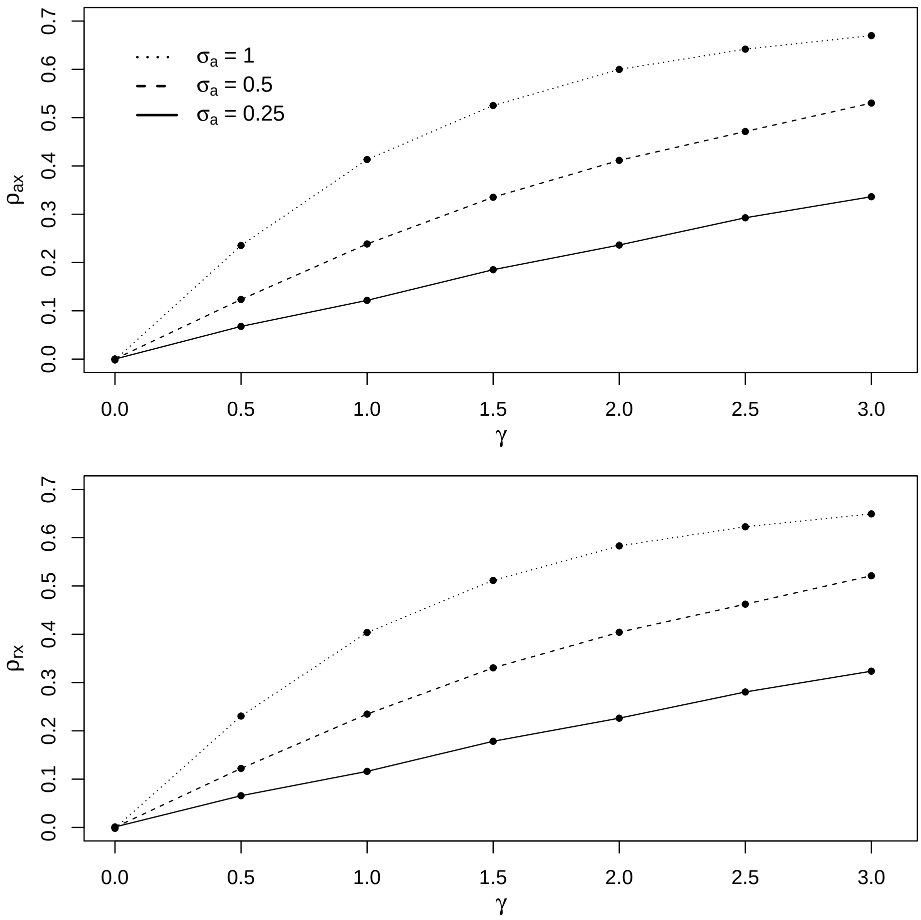

Figure 18: Correlations $\rho_{\mathbf{ax}}$ and $\rho_{\mathbf{rx}}$ for the simulated data with various values of γ.
